# Immune-focused RBD nanoparticles induce cross-reactive, RBS-directed responses capable of variant-resistant SARS-CoV-2 neutralization

**DOI:** 10.1371/journal.ppat.1013905

**Published:** 2026-02-19

**Authors:** Kylie M. Konrath, Kevin Liaw, Nicholas J. Tursi, Madison E. McCanna, Bryce M. Warner, Ebony N. Gary, Xizhou Zhu, Cory Livingston, Cara Monastra, Drew Frase, Neethu Chokkalingam, Alana Huynh, Nicholas Shupin, Kelly Bayruns, Sinja Kriete, Amber Kim, Joyce Park, Robert Vendramelli, Thang Truong, Kevin Tierney, Kimberly Azaransky, Estella Moffat, Carissa Embury-Hyatt, Lynn A. Beer, Oreoluwa Solanke, Susanne N. Walker, Richa Kalia, Hsin-Yao Tang, Laurent M.P.F. Humeau, Trevor R.F. Smith, Darwyn Kobasa, David B. Weiner, Daniel W. Kulp

**Affiliations:** 1 Vaccine and Immunotherapy Center, The Wistar Institute, Philadelphia, Pennsylvania, United States of America; 2 Perelman School of Medicine, University of Pennsylvania, Philadelphia, Pennsylvania, United States of America; 3 Special Pathogens Program, National Microbiology Laboratory, Public Health Agency of Canada, Winnipeg, Canada; 4 Vaccine and Infectious Disease Organization, University of Saskatchewan, Saskatoon, Canada; 5 Department of Biochemistry, Microbiology, and Immunology, University of Saskatchewan, Saskatoon, Canada; 6 National Center for Foreign Animal Disease (NCFAD), Canadian Food Inspection Agency, Winnipeg, Canada; 7 Ellen and Ronald Caplan Cancer Center, The Wistar Institute, Philadelphia, Pennsylvania, United States of America; 8 Inovio Pharmaceuticals, Plymouth Meeting, Pennsylvania, United States of America; National University of Singapore, SINGAPORE

## Abstract

New SARS-CoV-2 variants pose an ongoing threat due to persistent immune escape of natural and vaccine-induced immunity. The emergence of BA.1 (Omicron) produced a large antigenic shift in the spike protein, rendering many antibodies ineffective with concomitant loss of Emergency Use Authorization (EUA) status. While strains have evolved far from BA.1, re-emergence of variants from branches closer to BA.1 are of recent concern. Here, we engineered a self-assembling nanoparticle displaying RBD 4mut g5.1, an immunogen developed using structure-guided design to focus antibody responses to the receptor binding site (RBS) epitope and promote cross-reactivity by inclusion of four rationally selected BA.1 mutations in the RBS. Unlike multi-component RBD approaches, we demonstrate a single, rationally designed component is sufficient for generating broad immunity. We demonstrate that in both naïve and antigen-experienced mice, RBD 4mut g5.1 nanoparticle induced cross-reactive and durable antibody responses capable of potent neutralization of ancestral SARS-CoV-2 and many Omicron variants. RBD 4mut g5.1 provided heterologous protection at a memory timepoint. By showcasing how subtle changes in an epitope can trigger a diversified antibody response, this study offers a promising new avenue for developing vaccines that can more effectively tackle the ever-evolving threat of immune escape, not only against SARS-CoV-2 but potentially against a range of variable pathogens.

## Author summary

We showed that structure-guided design can be employed to rationally resurface epitopes on an immune-focused nanoparticle vaccine is a powerful strategy for eliciting broad and durable SARS-CoV-2 immunity.

## Introduction

New seasonal outbreaks fueled by emerging severe acute respiratory syndrome coronavirus 2 (SARS-CoV-2) variants continue to highlight the urgent need for next-generation vaccines that are resilient to antigenic drift. The World Health Organization (WHO) designates variants under monitoring (VUM), of interest (VOI), and of concern (VOC) to identify the viruses that pose the greatest present threats to public health [1]. Variants can arise from errors during replication, recombination [2,3], replication in immunocompromised individuals [4–6], and spillback events [7–9], posing an ongoing threat via immune evasion to vaccines and monoclonal antibody therapies [10–13]. In May 2021, VOC B.1.617.2 (Delta) emerged [14] with 8 mutations as well as deletions in the spike relative to ancestral, USA-WA1/2020 [15]. In November 2021, VOC B.1.1.529 (Omicron) emerged [14] and was the most dramatic antigenic drift yet, with over 30 substitutions in addition to deletions and insertions in the spike relative to USA-WA1/2020 [15]. Due to these events, vaccine efficacy against symptomatic Omicron infection dropped by nearly 30% relative to ancestral efficacy [16]. Strategies to mitigate the immune evasion of future viruses remain an important goal to protect from severe disease.

Early in the pandemic, receptor binding domain (RBD)-directed antibodies were determined to account for 90% of the neutralizing response [17] and have been a key focus of vaccine design. RBD-directed antibodies target four major epitopes, denoted classes 1–4 [18]. Classes 1 and 2 footprints partially overlap and target the receptor binding site (RBS), while classes 3 and 4 footprints recognize the RBD core [18]. Mutations in variants located in these four major epitopes contribute to immune evasion.

Omicron viruses have continued to diversify since the emergence of BA.1 [15,19]. In fact, VOC BA.1 (Omicron) has sixteen mutations in the RBD including eight mutations in class 1 and 2 epitopes. Thus, updated seasonal vaccines with new strains are constantly being developed. One study of a VOC XBB 1.5 vaccine reported a 71% vaccine effectiveness at preventing COVID-19 hospitalization in those over 60 years old, suggesting updating the strains included in vaccines can protect vulnerable populations [20]. Current Omicron vaccines can induce protective responses in part through boosting neutralizing antibodies from memory against conserved epitopes [21–24]. However, the development of vaccines that can induce broader immunity could circumvent the need to take seasonal VOC boosters. Significant efforts have been undertaken to induce broad SARS-CoV-2 responses such as through focusing antibody responses to conserved class 3 or class 4 epitopes [25–29]. While less effort has been dedicated to broadening class 1 and class 2 immune responses, there are cross-reactive class 1 and class 2 monoclonals that have been discovered [24,30] suggesting class 1 and 2 epitopes are also a promising target for broad SARS-CoV-2 immunity.

Immunogen design strategies such as glycan masking and epitope resurfacing can be used to influence the antibodies induced by vaccination. Proteins can be decorated with potential N-linked glycosylation sites (PNGS) in a technique called glycan masking. These engineered glycans can sterically shield the protein from antibodies to reduce responses to the epitope underneath and near the sugar and ultimately reduce responses to epitopes that are less relevant for protection [31–33]. To induce responses against immunosubdominant epitopes, immunogenic domains or patches can be replaced with novel sequences to influence antibody responses toward conserved epitopes [27,34,35]. Together, glycan masking and resurfacing may provide powerful tools to approach the changing landscape of SARS-CoV-2 viruses.

Here, we explored using structure-guided design to selectively incorporate Omicron mutations in an immunogen as an approach to improve cross-reactive, RBS-directed responses that broadly neutralize both ancestral and contemporary Omicron viruses. We carefully examined the RBS surface and mapped three datasets onto the class 1 and class 2 epitopes: structures of neutralizing antibodies (nAbs), frequency of viral mutations and viral indels. Our RBS-focused nanoparticle designs demonstrated cross-reactive protection in a heterologous B.1.617.2 (Delta) challenge and by incorporating just 4 Omicron mutations can provide near-sterilizing immunity against BA.2 (Omicron) challenge. We analyzed the antibody response by negative stain electron microscopy polyclonal epitope mapping (nsEMPEM) discovering a new class of antibody, ‘X1-2’, targeting the RBS. We showed that structure-guided design can be employed to rationally resurface epitopes on an immune-focused RBD nanoparticle as powerful strategy for eliciting broad and durable SARS-CoV-2 immunity, potentially reducing the need for frequent vaccine reformulations.

## Results

### Ancestral-based RBD g5.1 24mer exhibits cross-reactivity and protects against B.1.617.2 variant lethal challenge

**Fig 1:**
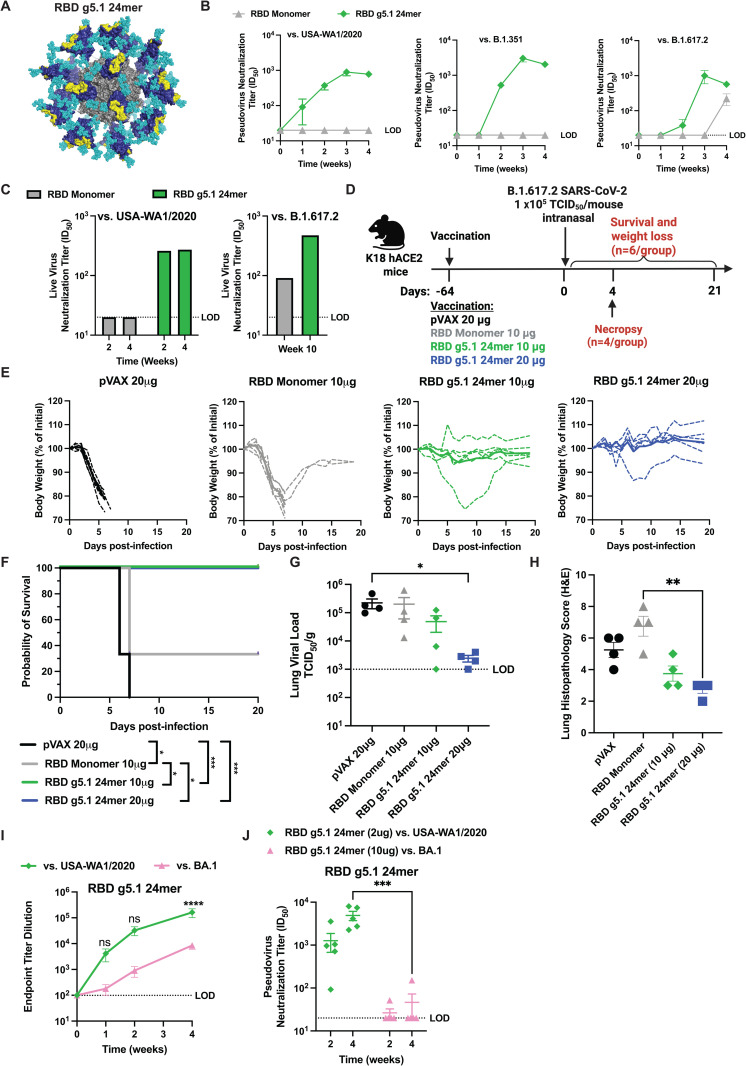
USA-WA1/2020 based RBD g5.1 24mer design induces cross-reactive responses through early SARS-CoV-2 variants and protects B.1.617.2 variant. A) Model of RBD g5.1 24mer nanoparticle where the surfaces for RBD, RBS, and ferritin scaffold are shown by surfaced colored dark blue, yellow, and gray respectively. Glycans are in cyan spheres. B) Pseudovirus endpoint titers to variant viruses of sera and C) live virus neutralization of sera from mice after single 2ug immunization (n=5 mice/group pooled sera). D) Immunization scheme for *in vivo* lethal B.1.617.2 (Delta) variant challenge, with single immunization at 10ug or 20ug, followed 64 days after by 1x10^5^ PFU intranasal B.1.617.2 challenge for data in E-H (n=10 mice/group). E) Weight loss curves from B.1.617.2 challenge. Individual mice represented by dashed lie and solid line is the average. F) Survival was monitored post-challenge. Survival was compared by Mantel-Cox log-rank test. G) Viral loads in lung tissue four days post-challenge and differences analyzed by Kruskal-Wallis test followed by post hoc Dunn’s analysis. H) Pathology scoring of H&E stained lung in challenged mice in subset of mice at day 4 post-challenge and compared by Kruskal-Wallis test followed by a post hoc Dunn’s analysis * p < 0.05, ** p < 0.01. LOD is limit of detection. Data in B and C were generated using pooled samples, so no statistical tests were run. Portions of this figure were created in BioRender Laenger, N. (2025) https://BioRender.com/s1hom96.

Previously, we developed RBD g5.1 24mer by incorporating PNGS into the ancestral USA-WA1/2020 RBD to focus responses toward the RBS epitope which is recognized by potently neutralizing class 1 and 2 antibodies [31] (Fig 1a). Upon the emergence of early VOCs, it was observed in human sera that there was a reduction in neutralization relative to the USA-WA1/2020 ancestral virus against B.1.351 (Beta), B.1.1.7 (Alpha), P.1 (Gamma), B.1.617.2 (Delta) [10,32–34] which had 1, 3, 3, and 2 mutations respectively in the RBS epitope [15]. We previously demonstrated that a single 5 µg dose of RBD g5.1 24mer induced cross-reactive responses to all these variants and were durable in mice for at least 6 months [31]. To further assess the potency of the observed cross-reactivity, we immunized BALB/c mice with a single 2 µg dose of DNA encoding RBD g5.1 24mer or USA-WA1/2020 RBD monomer. We observed that RBD g5.1 24mer elicited high neutralizing titers against B.1.351 (Beta), and B.1.617.2 (Delta) and autologous USA-WA1/2020 ancestral isolate (Fig 1b). Similar trends were observed for binding titers (S1a Fig). RBD monomer at the same dose induced weaker binding and minimal-to-no neutralizing titers to USA-WA1/2020, B.1.351 (Beta), P.1 (Gamma), and B.1.617.2 (Delta) (Figs 1b and S1a). Sera from RBD g5.1 24mer animals showed higher live virus neutralization than the RBD monomer vaccine sera of USA-WA1/2020 (ID_50_ > 250 vs below Limit of Detection) and B.1.617.2 (ID_50_ 474 vs 91) (Fig 1c).

To assess the functional protection against infection, 40 K18-hACE2 mice (n = 10 per group) were immunized with 20 µg of empty pVAX vector, 10 µg of RBD monomer, 10 µg of RBD g5.1 24mer, or 20 µg of RBD g5.1 24mer, followed by a lethal, heterologous B.1.617.2 challenge 64 days post-immunization (Fig 1d). Six of ten mice per group were monitored 21 days post-challenge for morbidity and mortality and the other 4 mice per group were necropsied at 4 days post-challenge for viral burden analysis (Fig 1d). The antigen-naïve, pVAX immunized mice succumbed to infection and rapidly lost weight in the first week post-challenge (Figs 1e and S1d). RBD monomer immunized mice succumbed to infection and rapidly lost weight within the first week, but two mice recovered by the end of the challenge (Fig 1e). In the RBD g5.1 24mer groups, only one mouse in the lower dose exhibited weight loss but recovered by the end of the challenge (Fig 1e). RBD monomer provided 33% protection, but remarkably both doses of RBD g5.1 24mer achieved 100% protection with just a single immunization, a statistically significant improvement over RBD monomer (Fig 1f). In the mice necropsied at day 4 post challenge, we observed lower viral loads within lungs in the RBD g5.1 24mer groups, with the high dose group exhibiting viral burdens near the limit of detection which was a significant reduction compared to pVAX group (Fig 1g). RBD g5.1 24mer at 20 µg dose significantly reduced the lung pathology compared to the other groups (Figs 1h and S1b-c). There was no statistically meaningful difference in disease burden between pVAX and RBD monomer groups by IHC and H&E of lung tissue or by viral titer in the lung (Figs 1g-h and S1b-c). The potent protection observed in heterologous B.1.617.2 challenge following RBD g5.1 24mer immunization demonstrate that immune-focused nanoparticles can maintain potency even against viruses that have some mutations in the epitope being targeted by antibodies (S1 Table).

### Immunogen is minimally resurfaced to improve Omicron variant-reactivity

**Fig 2:**
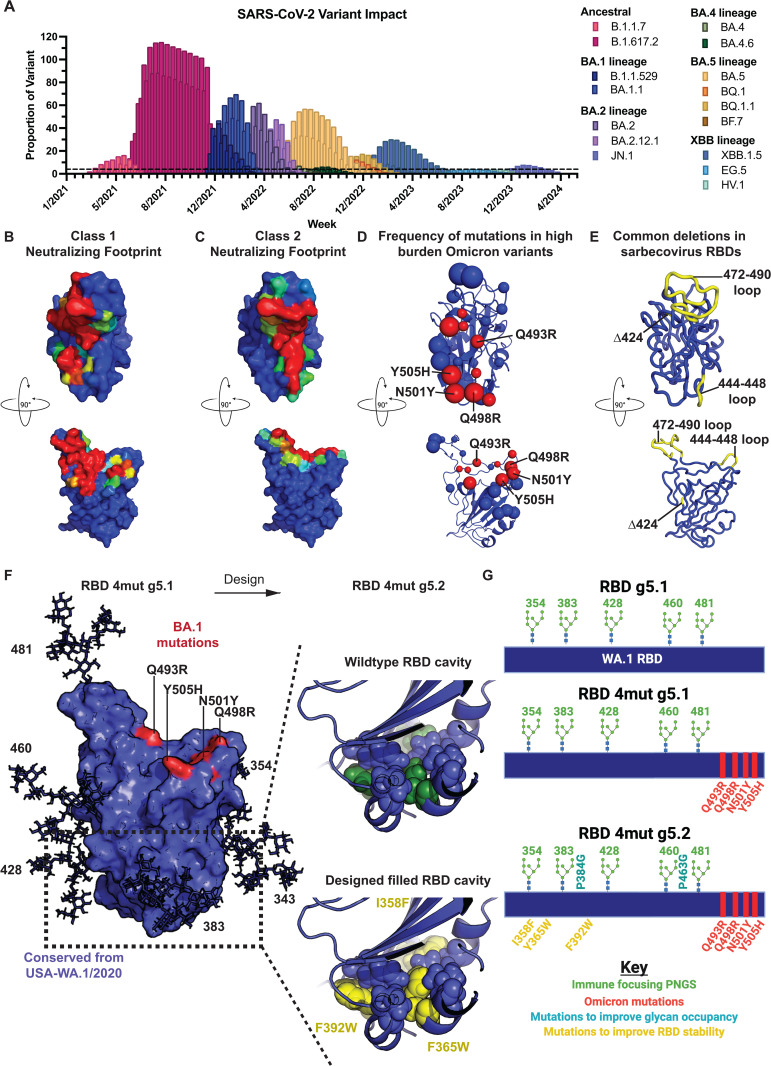
Bioinformatic analysis of the RBS epitope in Omicron variants. A) CDC variant proportion database entire U.S. dataset was sorted by variant over time. Variants that had at least a reported proportion of 4 for at least one reported week (above the line) are plotted. For B-E, top view of the RBD (top row) shows where ACE2 binds and side view of the RBD (bottom row). RBD surface colored to represent the most common positions contacted by B) class 1 and C) class 2 neutralizing antibodies. The surface is colored where red positions are most contacted by antibodies of the given class and blue is the least contacted by antibodies of the given class. D) Spheres represent the positions of mutations occurring in significant variants. Sphere size represents how many variants contain defining mutations (mutations that occur in >50% of sequenced viruses) at that position and red spheres are positions of mutations that are within 4Å of ACE2. Positions that have been mutated in variants but to multiple amino acid identities are not indicated with spheres. The four labeled mutations were used in immunogen design. E) Regions where deletions have been identified in non-human mammal (non-SARS-CoV-1 or -2) sarbecovirus RBDs are shown in yellow. F) RBD 4mut g5.1 immunogen has selective BA.1 mutations (red patches) and a set of glycans (sticks) to mask non-RBS epitopes. RBD 4mut g5.1 was stabilized in the RBD core to make the RBD 4mut g5.2. The numbers next to glycans indicate the position of the asparagine. G) Plasmid construction of immune focused monomer (top), epitope resurfaced modification (middle), and cavity filled-glycan occupancy modified (bottom). Portions of this figure were created in BioRender, Laenger, N. (2025) https://BioRender.com/s1hom96.

Despite the powerful cross-reactivity observed through B.1.617.2, RBD g5.1 24mer induced immunity exhibited decreased binding and significant loss of neutralization against BA.1 (Omicron) pseudovirus compared to USA-WA1/2020 (Fig 1i-j). Approved vaccines based on the ancestral isolates displayed similar losses in cross-reactivity [35–38]. BA.1 and subsequent Omicron variants contain considerably more mutations (up to 27) within the RBD than previous variants (S1 Table). Eight of the sixteen RBD mutations in BA.1 strain were in class 1 and 2 epitopes and explains the loss of neutralization by RBD g5.1 24mer vaccine sera since the RBS was being targeted by antibodies (S1 Table). New Omicron viruses have continued to emerge since the end of 2021 and contribute heavy proportions of disease in the U.S (Fig 2a).

To understand the potential impact of Omicron lineage mutations on class 1 and 2 antibodies, a structural analysis was performed to identify the most common positions contacted by class1 and class 2 neutralizing antibodies. Class 1 and 2 antibodies contact slightly overlapping surfaces near the RBS where ACE2 binds (Fig 2b-c). To understand the relative frequency of mutations in the most impactful Omicron viruses, we identified viruses with the heaviest burden on the U.S. using the U.S. Centers for Disease Control and Prevention (CDC) virus proportions database [39–41] (Fig 2a) and analyzed the major mutations for each variant (S1 Table). There were nine mutations in major Omicron variants (B.1.1.529-JN.1) that are within 4Å of ACE2 (shown as red spheres in Fig 2d). Four mutations that are frequently occurring in Omicron strains cluster together in one patch: Q493R, Q498R, N501Y, and Y505H (Fig 2d). In non-human, mammalian sarbecoviruses (excluding SARS-CoV-1 and -2), there are deletions in three portions of the RBD: 424, 444–448, 472–490 (Fig 2e). Deletions of 5 or more amino acids near the RBS occur in 444–448 and 472–490 loops (USA-WA1/2020 numbering) in >80% of sarbecoviruses (S2a Fig), resulting in important changes to the RBS backbone (S2b-c Fig). We identified 4 common mutations of Omicron—Q493R, Q498R, N501Y, and Y505H—that occur within the class 1 or 2 epitopes and cluster away from the less conserved 444–448 and 472–490 RBS loops that may be susceptible to deletion in future VOCs (Fig 2e-f). The four identified mutations can perturb monoclonal and polyclonal antibody binding confirming the validity of our structural analysis in identifying mutations that can antigenically resurface epitopes [10,42,43].

We incorporated these four Omicron mutations into an USA-WA1/2020-based RBD with the same PNGS used to immune focus in RBD g5.1 24mer construct to create a monomer, RBD 4mut g5.1 (Fig 2f). To further refine of our design for improved expression, folding, and immune focusing capacity, we mutated the cavity to stabilize the RBD and amino acids near designed PNGS to promote glycan occupancy in a design called RBD 4mut g5.2 (Fig 2f-g). Stabilizing mutations were selected from deep mutational scanning (dms) hits that improved stability and expression [44,45]. Three of the dms hits were used in the RBD 4mut g5.2 design—I358F, Y365W, F392W—to improve folding through Van der Waals forces in the core of the RBD (Fig 2f). Our previous work identified that PNGS at 383 and 460 were unoccupied in the RBD g5.1 24mer [31], perhaps due to prolines near the PNGS sequons [46,47]. Prolines at the i + 1 position of the 383 PNGS and an i + 4 position of the 460 PNGS were both mutated to glycine to promote occupancy in the RBD 4mut g5.2 design (Fig 2g).

**Fig 3:**
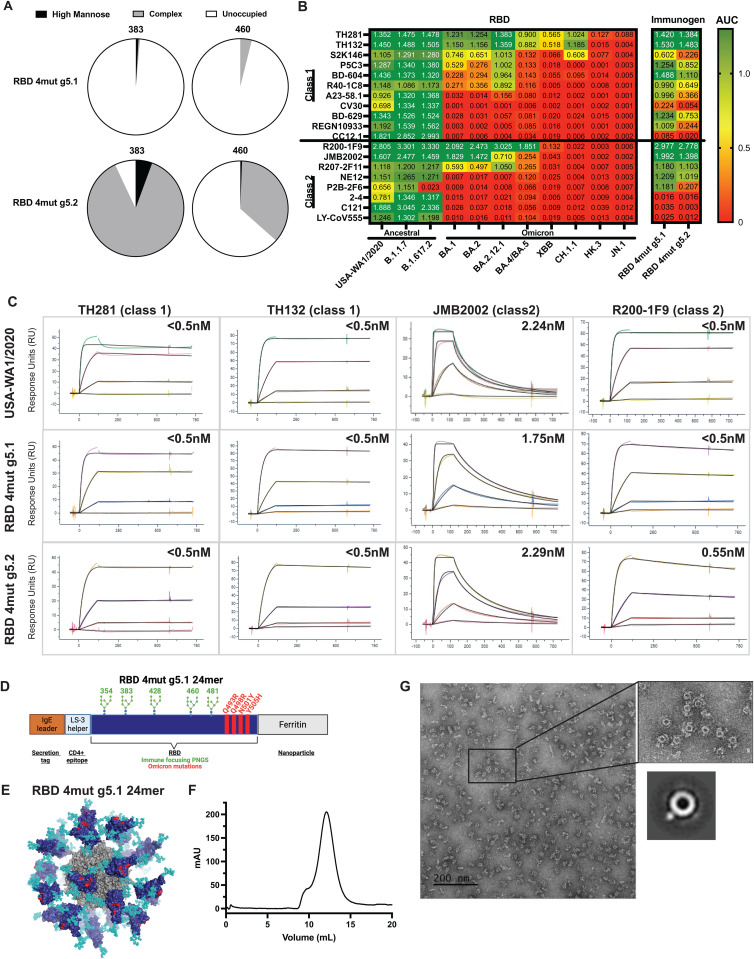
*In vitro* characterization of redesigned immunogens redesigned for cross-reactivity to ancestral and Omicron strains. A) Mass spectrometry analysis of glycan occupancy and species at each of the designed glycan positions. B) Heatmap of AUCs determined by ELISA for class 1 and 2 antibodies to immunogens and SARS-CoV-2 RBD antigens. C) Sensograms of most cross-reactive antibodies. KDs are corrected for glycan mass and occupancy and are indicated in the top right of each sensogram. D) Model of RBD 4mut g5.1 24mer nanoparticle where the surfaces for RBD, 4 mutations of BA.1, and ferritin scaffold are shown by surfaced colored dark blue, red, and gray respectively. Glycans are in cyan spheres. E) Size exclusion chromatography trace of RBD 4mut g5.2 24mer. F) On the left, a negative stain electron micrograph of RBD 4mut g5.2 24mer with a zoomed in view of nanoparticles. A representative 2D class average of the nanoparticle is to the bottom right.

We expressed and purified RBD 4mut g5.1 and RBD 4mut g5.2 to compare the designs biochemically and biophysically. We analyzed the glycan occupancy and species of the two designs. The occupancy of the RBD 4mut g5.1 design was consistent with RBD g5.1 as expected with the 383 and 460 positions being almost entirely unoccupied [31] (Figs 3a and S3). The RBD 4mut g5.2 had improved occupancy at 383 and 460 suggesting that the prolines near the PNGS were limiting occupancy and that g5.2 may improve immune focusing (Fig 3a).

We compared binding of a panel of class 1 and 2 monoclonal antibodies against USA-WA1/2020 RBD, our 4mut immunogens, and a panel of variant RBDs [30,48–62]. The level of cross-reactivity of the antibodies against USA-WA1/2020 through JN.1 occurred on a spectrum for both class 1 and 2 antibodies, but the binding was completely lost for all monoclonals tested against HK.3 and JN.1 (Fig 3b) and was consistent with the continued antibody escape observed in human sera and with monoclonal antibodies [30,63]. Three of the least broad class 1 and class 2 antibodies in our set (CC12.1, 2–4, C121, and LY-CoV555) had minimal interaction with RBD 4mut g5.1 and RBD 4mut g5.2 which is suggestive that these antibodies are particularly sensitive to the four Omicron mutations selected in the design (Fig 3b) and is consistent with the reported restricted reactivity of these antibodies [30]. RBD 4mut g5.1 and RBD 4mut g5.2 bound well to the majority of class 1 and 2 antibodies in the panel displaying intermediate binding between USA-WA1/2020 and BA.1 (Fig 3b). Interestingly, RBD 4mut g5.1 and g5.2 displayed strong binding to the most cross-reactive antibodies for class 1 (TH281, TH132) and class 2 (R200-1F9, JMB2002). We measured affinities using SPR for class 1 and 2 antibodies for both the RBD 4mut g5.1 and RBD 4mut g5.2 (Fig 3c). The most cross-reactive antibodies showed comparable nanomolar or higher affinity for USA-WA.1/2020, RBD 4mut g5.1 and RBD 4mut g5.2 (Fig 3c).

To enhance the potency, breadth, and durability of our immunogens we engineered nanoparticles to display RBD 4mut g5.1 and g5.2 (Fig 3d). The transgene contained an IgE leader to promote expression and secretion followed by the LS-3 CD4 helper epitope [64] with a glycine-serine linker to the RBD immunogen which was connected to ferritin (24mer) scaffold (Fig 3d). We transfected RBD 4mut g5.1 24mer and RBD 4mut g5.2 24mer nanoparticle designs, harvested supernatant, and purified supernatant by lectin affinity chromatography then further refined by size exclusion chromatography [SEC). A representative SEC trace for RBD 4mut g5.2 24mer showed a homogenous peak eluting at the expected size (Fig 3f). The nanoparticle was assessed by negative stain electron microscopy and we observed a central ring-like scaffold decorated by small densities, consistent with the design of ferritin displaying multiple RBDs [65] (Fig 3f).

### Resurfaced immunogen achieves potent, cross-reactive immunogenicity

**Fig 4:**
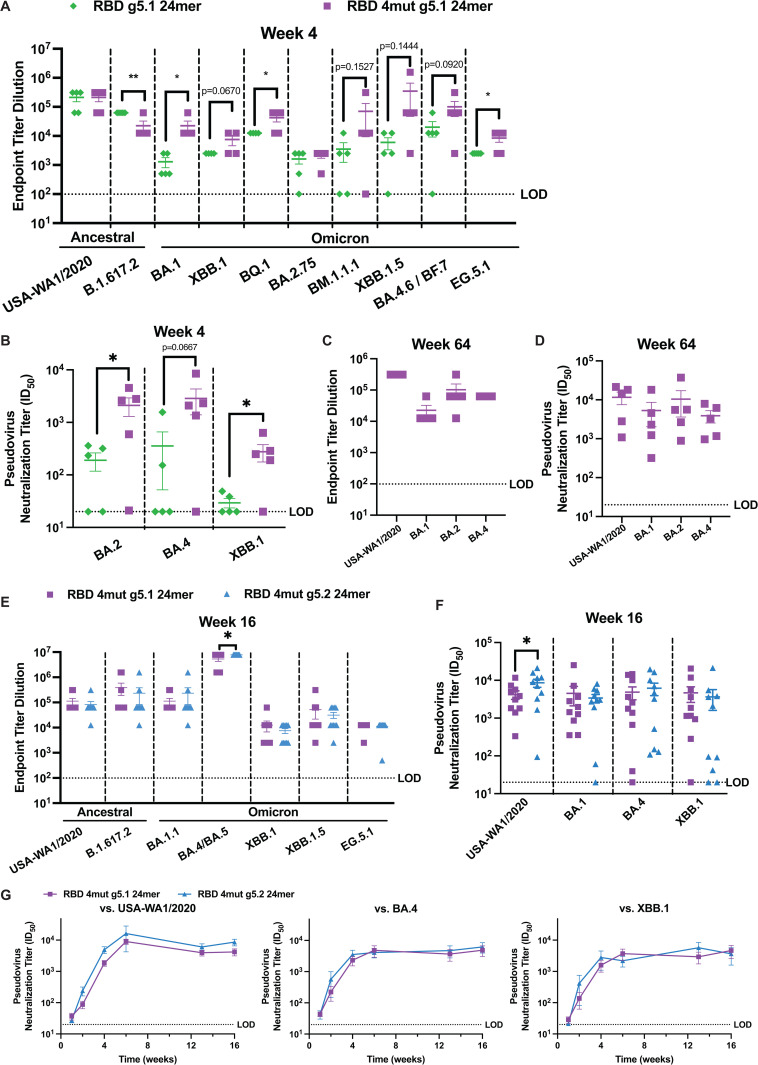
Comparison of immunogenicity of next generation Omicron RBD nanoparticle designs *in vivo.* A) Binding endpoint and B) pseudovirus neutralization titers 4 weeks after 10ug immunization of BALB/c mice with RBD 4mut g5.1 24mer or RBD g5.1. C) Binding endpoint and D) pseudovirus neutralization titers 64 weeks after 10 ug immunization of RBD 4mut g5.1 24mer. For A-D, n=5 mice/group. E) Binding endpoint and F) pseudovirus neutralization titers 16 weeks after 10 ug immunization of RBD 4mut g5.1 24mer or after 10 ug immunization of RBD 4mut g5.2 24mer. G) Pseudovirus neutralization titers over time following 10 ug immunization of RBD 4mut g5.1 24mer vs. RBD 4mut g5.2 24mer. For E-G, n=10 mice/group. For A-B and E-F, one-tailed t tests were performed to compare responses between vaccines for each variant. For G, differences between vaccines were assessed by Šídák multiple comparisons. * p < 0.05, ** p < 0.01. LOD is limit of detection.

To compare differences in immunogenicity to our original RBD g5.1 24mer design, BALB/c mice were immunized with a single 10 µg dose of a DNA plasmid encoding RBD g5.1 24mer or RBD 4mut g5.1 24mer and we analyzed binding and neutralization at week 4. Given that lung viral titer data following B.1.617.2 challenge were comparable between 10 µg and 20 µg of RBD 5.1 24mer, we were uncertain if a 20 µg dose would enable the power for detecting a statistically meaningful difference between the RBD g5.1 24mer and RBD 4mut g5.1 24mer designs, so a 10 µg was selected for immunogenicity comparisons. A 10 µg dose may also be more preclinically protective from morbidity than smaller doses we have explored [36]. Compared to RBD g5.1 24mer, RBD 4mut g5.1 24mer induced comparable binding titers to USA-WA1/2020 RBD and high, albeit slightly lower binding titers against B.1.617.2 (Fig 4a). Interestingly, RBD 4mut g5.1 24mer induced equivalent or greater binding titers to subsequent Omicron sublineages through EG.5.1 as compared to RBD g5.1 24mer (Fig 4a). The neutralizing response is significantly higher for mice immunized with RBD 4mut g5.1 24mer than mice immunized with RBD g5.1 24mer across all the Omicron variants we measured (Figs 4b and S4a-b). Notably, RBD 4mut g5.1 24mer immunized-mice maintained breadth and neutralization against USA-WA1/2020, BA.1, BA.2, and BA.4 through at least week 64 post-immunization, indicating durable and potent antibody responses (Fig 4c-d).

We next compared the immunogenicity of RBD 4mut g5.1 24mer and RBD 4mut g5.2 24mer. To explore the difference in immunogenicity due to RBD stability and glycan occupancy, we immunized BALB/c mice with a single 10 µg dose of DNA encoding RBD 4mut g5.1 24mer or RBD 4mut g5.2 24mer and analyzed week 16 serology for breadth. Strong but equivalent binding titers to all Omicron RBDs was observed after both vaccines with only a significant improvement against BA.4/BA.5 in the RBD 4mut g5.2 24mer group, suggesting no global differences in humoral responses (Figs 4e and S3b-c). Neutralization titers to variants were similarly equivalent at all time points measured up to week 16 (Fig 4f-g). The data here supports that minimal resurfacing of an epitope can simultaneously induce both ancestral and contemporary SARS-CoV-2 immunity, but that stabilization of the RBD does not globally improve immunogenicity. Given that there was reduction in binding *in vitro* of some class 1 and 2 monoclonal antibodies including S2K146, A23-58.1, CV30, REGN10933, and BD-629 to RBD 4mut g5.2 relative to RBD 4mut g5.1 (Fig 3b) and there no global statistical difference *in vivo* between RBD 4mut g5.1 24mer and RBD 4mut g5.2 24mer, we continued further *in vivo* characterization of the RBD 4mut g5.1 24mer.

### RBD 4mut g5.1 24mer protects in heterologous BA.2 memory challenge

**Fig 5:**
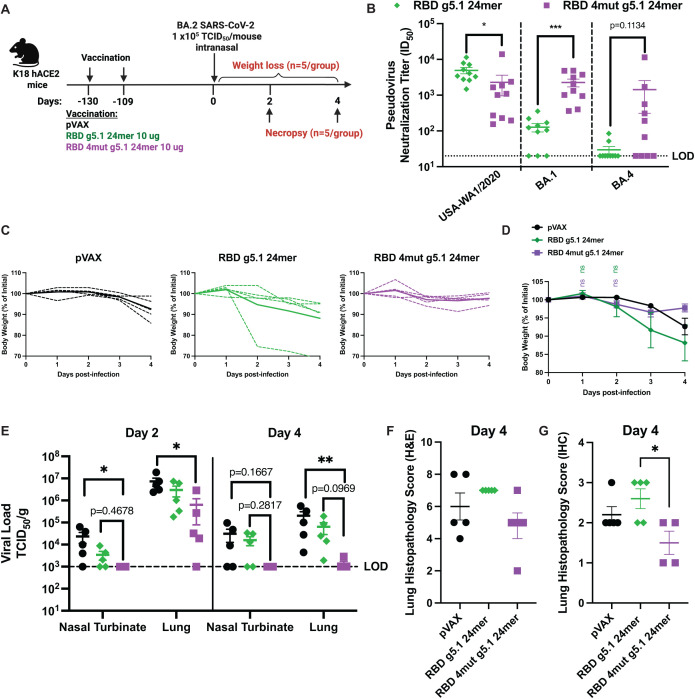
RBD 4mut g5.1 24mer protects mice in BA.2 memory challenge model. A) Immunization scheme of K18 hACE2 mice with two 10ug doses followed 109 days after boost with intranasal infection with BA.2 at 1x10^5^ PFU (n=10 mice/group). B) Pseudovirus neutralization from pre-challenge sera. C) Weight loss curves from BA.2 challenge. D) TCID_50_ of lungs and nasal turbinates from BA.2 challenge 2 and 4 days after challenge. E-F) Lung pathology scoring at day 4 post-challenge. * p < 0.05, ** p < 0.01. LOD is limit of detection. For B, one-tailed t tests were performed to compare responses between vaccines for each variant. For D-F, metrics of viral burden were compared by Kruskal-Wallis tests followed by a post hoc Dunn’s analysis. Portions of this figure were created in BioRender, Laenger, N. (2025) https://BioRender.com/s1hom96.

To evaluate the protection afforded from long-term immune memory, K18 hACE2 mice were immunized twice with 10 µg of pVAX, RBD g5.1 24mer, or RBD 4mut g5.1 24mer and performed a non-lethal challenge with BA.2 virus >100 days after boost (Fig 5a). In pre-challenge sera, RBD g5.1 24mer induced higher pseudovirus neutralization titers than RBD 4mut g5.1 24mer against USA-WA1/2020, but RBD 4mut g5.1 24mer induced higher neutralization against BA.4 and significantly higher BA.1 neutralization than RBD g5.1 24mer (Fig 5b). After infection, pVAX and RBD g5.1 24mer-immunized mice exhibited mild weight loss while RBD 4mut g5.1 24mer ameliorated this (Fig 5c-d). Relative to pVAX, RBD g5.1 24mer slightly reduced viral loads in the lungs and nasal turbinates and RBD 4mut g5.1 24mer significantly reduced viral loads (Fig 5e). Notably, RBD 4mut g5.1 24mer reduced viral loads within nasal turbinates below our limit of detection by day 2 post-infection and in both nasal turbinates and lungs by day 4 post-infection. Like the B.1.617.2 challenge, lung pathology was scored in a subset of mice at day 4 post-BA.2 challenge. Overall lung pathology scores were reduced in the RBD 4mut g5.1 24mer group compared to RBD g5.1 24mer (Fig 5f-g). The BA.2 memory challenge demonstrated that RBD 4mut g5.1 24mer immunity protects from disease burden of heterologous virus, even at a memory timepoint.

### RBD 4mut g5.1 24mer boosts cross-reactive responses after COVID-19 immunization

**Fig 6:**
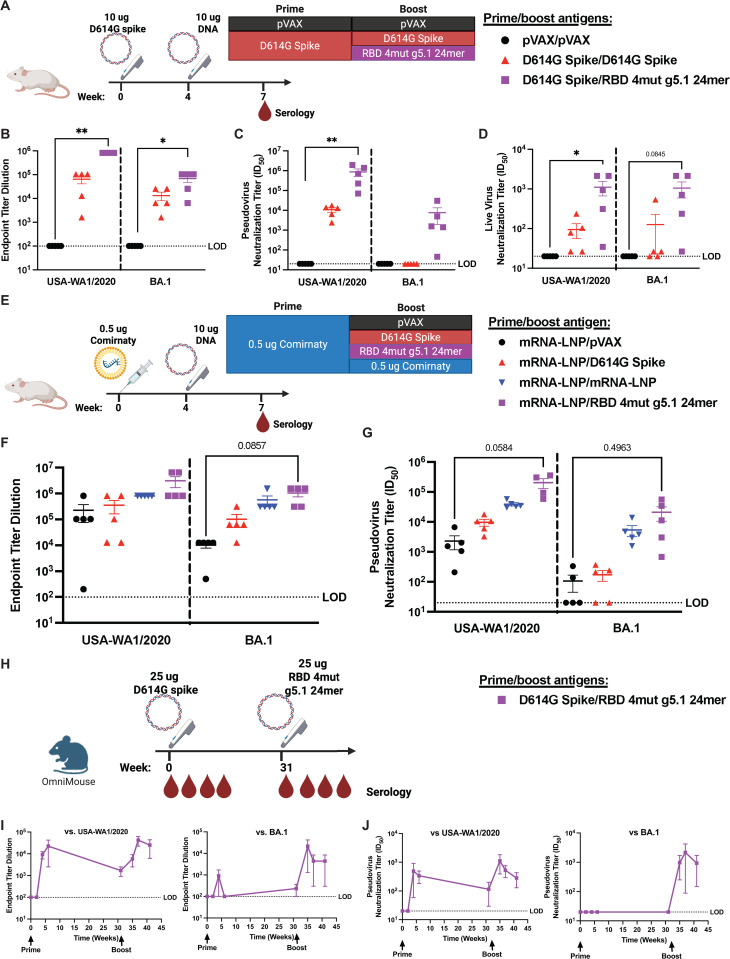
Immune focused nanoparticles effectively boost vaccine responses against variants in antigen experienced mice. A) Schematic of boosting DNA spike antigen experienced BALB/c mice for B-D (n=5 mice/group). B) Binding, C) pseudovirus neutralization, and D) live virus neutralization of DNA spike antigen experienced BALB/c mice. Labels denote boosting group after DNA Spike prime. E) Schematic of boosting Comirnaty mRNA antigen experienced BALB/c mice for F-G (n=5 mice/group). F) Binding and G) pseudovirus neutralization of Comirnaty mRNA antigen experienced BALB/c mice. Labels denote boosting group after Comirnaty mRNA prime. H) Schematic of boosting DNA spike antigen experienced human antibody repertoire mice for I-J (n=3 mice). I) Binding and J) pseudovirus neutralization of DNA spike antigen experienced human antibody repertoire mice. LOD is limit of detection. For B-D and F-G, differences between vaccination regiments were assessed by Kruskal-Wallis tests followed by a post hoc Dunn’s analysis. Portions of this figure were created in BioRender, Laenger, N. (2025) https://BioRender.com/s1hom96.

To evaluate the performance of RBD 4mut g5.1 24mer as a booster in mice with pre-existing SARS-CoV-2 spike immunity, mice were primed with 10 µg of plasmid DNA encoding D614G SARS-CoV-2 spike and boosted 4 weeks later with 10 µg of DNA plasmid encoding D614G SARS-CoV-2 spike or RBD 4mut g5.1 24mer. A group of control mice were primed and boosted with an empty pVAX vector (Fig 6a). We observed that boosting with RBD 4mut g5.1 24mer induced significantly greater antibody responses compared to naïve mice in terms of binding, pseudovirus neutralization, and live virus neutralization against ancestral USA-WA1/2020 RBD (Fig 6b-d). Interestingly, D614G spike immunized mice produced binding antibodies to BA.1 RBD (Fig 6b), but those antibodies did not neutralize BA.1 pseudovirus (Fig 6c). On the other hand, RBD 4mut g5.1 24mer immunized mice elicited BA.1 binding and neutralizing antibodies (Fig 6b-d). RBD 4mut g5.1 24mer vaccine also induced stronger binding and neutralization across Omicron sublineages through EG.5.1 compared to D614G spike boosted mice (S5a-b Fig).

To simulate immunity in the mRNA-LNP-immunized population, we primed BALB/c mice with a low-dose 0.5 µg of Comirnaty SARS-CoV-2 vaccine (similar dosing to previous studies [66]) and heterologously boosted with 10 µg of pVAX, D614G spike, or RBD 4mut g5.1 24mer. A control group was boosted with 0.5 µg of Comirnaty (Fig 6e). Boosting with RBD 4mut g5.1 24mer induced stronger binding and significantly stronger neutralization titers against both USA-WA1/2020 and BA.1 compared to pVAX boosting (Fig 6f-g). Relative to Comirnaty boosting, RBD 4mut g5.1 24mer improved binding titers against both USA-WA1/2020 and BA.1 as well as neutralization against BA.1 and almost significantly improved USA-WA1/2020 neutralization (Fig 6f-g). We observed binding titer improvements of the RBD 4mut g5.1 24mer booster compared to all the other booster vaccines including Comirnaty against a set of Omicron subvariants (S5c-d Fig).

Finally, we assessed RBD 4mut g5.1 24mer as a booster to mice with existing immunity from vaccination with D614G spike in the Omni mouse model which has humanized immunoglobulin loci with human V, D, and J gene segments [67] as this might better recapitulate the existing antibody lineages in humans. Omni mice were primed with 25 µg of DNA encoding the full D614G spike and boosted 31 weeks later with 25 µg of DNA encoding RBD 4mut g5.1 24mer (Fig 6h). Prior to boost, antibody binding and neutralization were poorly cross-reactive to BA.1 and had waned against matched USA-WA1/2020 since peak responses (Fig 6i-j). After RBD 4mut g5.1 24mer boost, responses to ancestral USA-WA1/2020 were boosted and novel cross-reactive responses to BA.1 were conferred. This shows that human VDJ antibody configurations can be elicited by RBD 4 mut g5.1 24mer that are cross-reactive between ancestral and the BA.1 variant.

Robust humoral responses were observed among SARS-CoV-2 antigen-experienced BALB/c mice following RBD 4mut g5.1 24mer immunization regardless of the initial COVID-19 vaccine delivery platform (mRNA-LNP or DNA). The data in the antigen-experienced experiments support that RBD 4mut g5.1 24mer would effectively induce broad, variant resistant immunity in antigen-experienced individuals. Assessing the differences, a memory timepoint rather than an acute one may better differentiate the response between boosting with USA-WA1/2020 spike and boosting with nanoparticle. The data in the waning immunity experiment in human antibody repertoire mice supports that RBD 4mut g5.1 24mer can quickly recall USA-WA1/2020 antibodies from memory and facilitate their development into cross-reactive responses.

### Antibody diversity confers improved breadth

**Fig 7:**
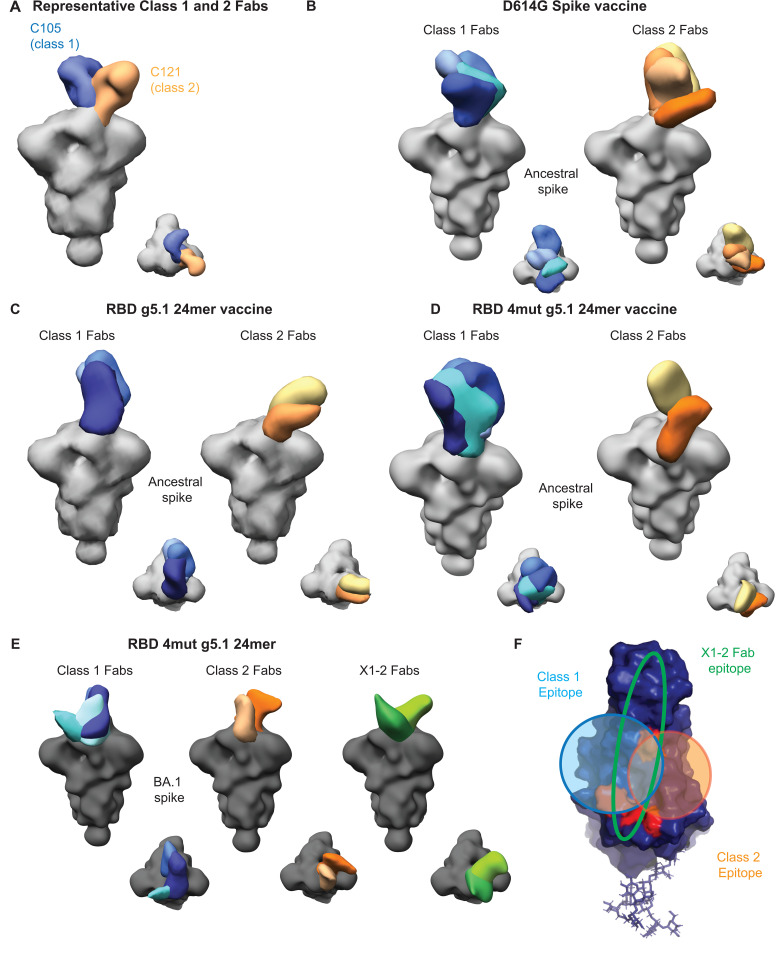
Vaccine induced antibodies targeting the RBD. **A)** Representative Fabs for class 1-C105 blue (EMDB 22128) and class 2- C121 orange (EMDB 22735) on a gray spike. Week 6 terminal sera pooled from BALB/c mice was tested against USA-WA1/2020 spike following 10 ug DNA immunization of B) spike **C)** RBD g5.1 24mer **D)** RBD g5.1 4mut 24mer (n = 10 mice/group). Representative Fabs recognizing BA.1 spike following immunization with **E)** RBD g5.1 4mut 24mer. In A-E, class 1-directed Fabs are colored blue, class 2-directed Fabs are orange/yellow, USA-WA1/2020 spike is light gray, and BA.1 spike is dark gray; side and top views of the spike-Fab complexes are shown. **F)** Summary of class 1, 2, and X1-2 epitopes on the 4mut RBD.

To compare antibody specificity as a potential mechanism for breadth, we immunized ten BALB/c mice once with 10µg of DNA encoding spike, RBD g5.1 24mer, or RBD 4mut g5.1 24mer and collected a terminal bleed after six weeks to visualize antibody specificity with electron microscopy polyclonal epitope mapping (EMPEM). The EMPEM technique isolates antibodies from sera samples, digests antibodies into Fabs, and then complexes Fabs with antigen for electron microscopy image collection and processing to reconstruct 3D structures of Fab-antigen complexes [68,69]. We created representative low-resolution densities using class 1 (C105) and 2 (C121) Fab structures in complex with spike to assign putative class 1 and 2 binding to Fabs found in the mouse sera (Fig 7a) [18].

First, we complexed Fabs from three different immunization groups with an ancestral (USA-WA1/2020) spike protein. In the spike-immunized group, eight unique Fab densities against the RBS were observed (Fig 7b), consistent with observations that the RBS is immunodominant in vaccine sera following DNA full spike immunization [70]. Four Fabs bound to the class 1 epitope and four Fabs bound to class 2 epitope (Fig 7b). We observed five RBD g5.1 24mer vaccine elicited antibodies that bound to ancestral spike at both the class 1 and class 2 epitopes, but were found to be more restrictive in the angle of approach relative to D614G Spike elicited antibodies (Fig 7b-c). In the RBD 4mut g5.1 24mer group, six unique densities were observed against the RBS, with four class 1 Fabs and two class 2 Fabs were observed (Fig 7d). The angles of approach for class 1 Fabs are more restricted than those observed in D614G immunizations, however they are slightly less confined than we observe with RBD g5.1 24mer antibodies.

Next, we purified complexes of RBD 4mut g5.1 24mer Fabs with a BA.1 variant spike to compare the BA.1 and ancestral antibody specificities. We observed four class 1 Fabs and two class 2 Fabs (Fig 7e). Interestingly, we observed two Fabs with unique angles of approach in complex with the BA.1 spike that approached at opposing angles across the RBS in a novel way across the class 1 and 2 epitopes (denoted X1-2 Fabs) (Fig 7f). One of the X1-2 Fabs appeared to straddle the two sides of the RBS from at an angle from the exterior of the spike (dark green Fig 7f). The other X1-2 Fab approached at an angle from the center part of the spike more parallel with the top of the trimer than observed in the other samples (light green Fig 7f). These novel X1-2 antibodies may not be cross-reactive to USA-WA1/2020 (Fig 7d) as they do not appear in the USA-WA1/2020 complex (Fig 7d). Further investigation of X1-2 antibodies could be performed to understand their contribution to the observed breadth across Omicron sublineages. EMPEM with BA.1 spike was not performed for the spike and RBD g5.1 24mer groups given that the groups had minimal neutralization and the affinity was likely too weak to robustly purify complexes [30]. Higher resolution structures and monoclonal isolation could provide additional insights on the mechanism of antibody lineages achieving breadth.

## Discussion

SARS-CoV-2 vaccine campaigns prevented an estimated 18 million deaths from COVID-19 in the first year of their deployment alone [71] and have continued to reduce hospitalization risks against variants [72], yet waves of infection continue. Omicron sublineages limit cross-reactivity of previously induced antibodies through the introduction of 8 mutations within the class 1 and 2 epitopes relative to the ancestral USA-WA1/2020 sequence [30,48–56]. We developed a new analysis by collating the structural footprints of nAbs with mutations most frequently observed in SARS-CoV-2 variants. This analysis led us to focus on 4 RBS mutations—Q493R, Q498R, N501Y, and Y505H—that occur frequently in Omicron sublineages. These positions are commonly located in the footprints of class 1 and 2 antibodies and we hypothesis could form a surface patch to stimulate cross-reactive antibodies. Given the observed reversion mutations from Omicron back to ancestral strains, and the possibility of zoonotic spillover reintroducing ancestral-like viruses [7–9], we developed RBD 4mut g5.1/g5.2 24mer using the ancestral RBD sequence with the 4 RBS mutations engineered to broaden SARS-CoV-2 responses to include immunity to both ancestral and Omicron strains.

The antigenic and immunologic impacts of these mutations on an immune focused ancestral RBD are highly significant. RBD 4mut g5.1 bound with high affinity to many neutralizing antibodies that bind ancestral variants and to the most cross-reactive monoclonal antibodies. RBD 4mut g5.1 24mer induced strong titers to USA-WA1/2020 and cross-reactive binding and neutralization against all Omicron viruses tested. Antibodies induced by RBD 4mut g5.1 24mer were highly durable as we observed both potent heterologous BA.2 protection at an immunological memory timepoint of 100 days post-immunization and high neutralization titers against BA.1 for over a year after a single immunization. RBD 4mut g5.1 24mer was able to induce cross-neutralizing antibodies in antigen-experienced animal, such as those primed with the Comirnaty mRNA-LNP vaccine. The robust humoral responses in SARS-CoV-2 antigen-inexperienced mice suggests that this immunogen is promising for effective induction of broad, more variant-resistant immunity in naïve individuals. Further, RBD 4mut g5.1 was able to induce cross-reactive immunity in human antibody repertoire mice demonstrating our vaccine can elicit human antibodies that neutralize both USA-WA1/2020 and BA.1. EMPEM analysis of RBD 4mut g5.1 24mer immunized mice identified class 1 and 2 antibodies with similar angles of approach against both USA-WA1/2020 and BA.1 antigens suggesting that we have elicited some cross-reactive clones. RBD 4mut induced unique X1-2 antibodies that cross over the RBS at angles not described by any previous vaccine. Further exploration of X1-2 antibodies to understand their contribution to polyclonal Omicron breadth is needed to understand their immunological importance.

Interestingly, more contemporary viruses such as JN.1 and KP.2 contain L452W and L455S which are in the RBS and the resurfaced patch. Affinity and neutralization of class 1 antibodies is reported to be weakened to these more recent strains relative to early Omicron strains [81,82], but the capacity of class 2 and X1-2 antibodies to combat these more recent strains is an exciting future direction. Mapping atomic contacts made polyclonal antibodies induced by RBD 4mut g5.1 24mer such as by cryo-EM or antibody isolation are future avenues to explore the potential for antibody escape.

The prevailing strategy of using RBD-based vaccines to achieve broad SARS-CoV-2 immunity is highly limited by the need to encode multiple RBDs. Mosaic RBD nanoparticles that co-present multiple diverse clades of sarbecoviruses or variants of SARS-CoV-2 on the same nanoparticle have been explored for their ability to engage B cells that bivalently bind conserved epitopes on diverse neighboring RBDs and improve breath [25–27]. Despite these impressive results, multi-component vaccines face significant challenges that can limit their development and widespread use including complexities with formulation, manufacturing costs, regulatory hurdles, immunological competition between the antigens and less control of resulting immune response. Here, we achieved cross-reactive immunity through a simplified system of a single nanoparticle harboring one epitope-resurfaced and immune-focused RBD. Our findings provide a blueprint for developing broadly protective vaccines against other pathogens using epitope focusing and resurfacing that creates novel restrictions on pathogen immune escape.

## Materials and methods

### Ethics statement

All studies were performed at Wistar Institute animal facilities in accordance with approved protocols under Institutional Animal Care and Use Committees (Protocol 201399).

### Animals

BALB/c, C57BL/6, and K18-hACE2 mice were obtained from Jackson Laboratories. Animals were housed in ventilated cages and given free access to food and water. For DNA immunizations, plasmids were formulated in water and administered intramuscularly into the tibialis anterior muscle. Electroporation was then performed using CELLECTRA-EP delivery system consisting of two pulse sets at 0.2A of 52ms pulses and 198ms delay with 3 second interval. For RNA immunizations, Pfizer-BioNTech mRNA COVID-19 vaccine was obtained from the pharmacy, formulated in water, and administered intramuscularly into the tibialis anterior muscle. Blood was collected via submandibular bleed at specified time points for serology.

### ELISA

For analysis of mouse serum samples, high binding 96-well flat-bottom half-area microplates (Corning) were coated overnight at 4°C with 1μg/mL of SARS-CoV2 RBD of variants (Sino Biological). Plates were then blocked for 2 hours at ambient temperature in PBS with 5% Milk, 0.2% Tween-20. Serial dilutions of sera were made and applied to the plates, followed by 2 hours incubation at 37°C. Plates were then incubated for 1 hour at ambient temperature. For BALBc and K18-hACE2 mouse studies, secondary antibody goat anti-mouse IgG h + l HRP-tagged antibody (Bethyl Laboratories) was used at a 1:20,000 dilution. For Omni mouse studies, goat anti-human κ + λ light chain HRP-tagged antibody was used at a 1:10,000 dilution. All dilutions were made in PBS with 1% NCS, 0.2% Tween-20, and plates were washed in between steps in PBS with 0.05% Tween-20 with a 405LS automated plate washer (Biotek Instruments). Plates were then developed with 1-step Ultra TMB substrate (Thermofisher) for 5 minutes and quenched with 1N H_2_SO_4_ solution. Plates were read on a Synergy 2 plate reader (Biotek Instruments) at 450 and 570 nm absorbances.

### Surface plasmon resonance

RBD-antibody kinetics experiments were performed with a Series S Sensor Protein A capture chip (Cytiva) on a Biacore 8k instrument (GE). HBS-EP+ running buffer was used (Teknova). Each experiment began with two start up cycles with 60 s of contact time and a flow rate of 50 mL/min. For analysis methods, approximately 150–250 RUs of IgG antibodies was captured on each flow cell at a flow rate of 10 mL/min for 60 seconds. RBD or glycan variants samples were 6x serial diluted from 200 nM (based on theoretical mass of the RBD without glycans) in running buffer and flowed across the chip after capture at a 50 mL/min rate. The experiment had a 120 second contact time phase and a 600 second dissociation phase. Regeneration was performed with 10 mM glycine at pH = 1.5 at a flow rate of 50 mL/min for 30 seconds after each cycle. Results were analyzed with 1:1 kinetic fitting in the Biacore Insight Evaluation software version 5.0.18.22102 (Cytiva).

For KD calculations, molecular weights of proteins were adjusted to include the mass contributed by glycans. The molecular weight of glycans in the RBD g5.1 monomer design was previously determined by SEC MALS as 12.27 kDa [31]. The occupancy determined here for RBD 4mut g5.1 monomer was an average of 3.75 glycans and assumed to be approximately equivalent to the RBD g5.1 design. Based on SEC MALS data, this was approximately 3.27 kDa per glycan. The number of average glycans on the RBD as determined by mass spectrometry analysis for RBD 4mut g5.1 or RBD 4mut g5.2 and this 3.27 kDa mass per glycan was used to determine the molecular weight of the RBD and concentration range used in the experiment. Average occupancy of native glycans for positions 331 and 343 for the RBD 4mut g5.1 and RBD 4mut g5.2 designs were used to estimate the molar mass of variant RBDs.

### Pseudovirus generation and neutralization assay

HEK293T cells were obtained from ATCC. CHO-hACE2 cells were obtained from Creative Biolabs. Cells were maintained in DMEM supplemented with 10% heat-inactivated fetal bovine serum (FBS) and 1% penicillin-streptomycin (P/S) antibiotic at 37°C under 5% CO_2_. To generate SARS-CoV2 spike containing pseudoviruses for luciferase-based virus neutralization assays, HEK293T cells were seeded at 5 million cells in T75 flasks and grown overnight. Cells were then treated with 48μL GeneJammer (Agilent), 6μg S_IgE_deltaCterm19 pseudovirus plasmid (Genscript), and 6μg pNL4–3.luc.R-E- backbone (Aldevron). For variants, cells were treated similarly but instead with variant pseudovirus plasmids. Transfection supernatants were collected 72 hours later and supplemented with 12% FBS. Pseudovirus solutions were stored at -80°C until further use. Pseudovirus solutions were titered to determine dilutions for working solutions such that they yielded >15-fold greater relative luminescence compared to cells alone control.

For pseudovirus assays, CHO-hACE2 cells were seeded in 96-well plates at 10,000 cells/well and incubated overnight. Sera from vaccinated mice were heat inactivated for 15 min at 56°C, then diluted in DMEM with 10% FBS and 1% P/S. Virus solutions at the determined working titer were then introduce to the dilutions and incubated for 90 min at ambient temperature. This media containing diluted sera and pseudovirus was then applied to CHO-hACE2 cells and incubated for 72 hrs. Plates were developed using the BriteLite plus luminescence reporter system (Perkin Elmer) and read on a plate reader. Percent neutralization was calculated based on virus only positive control signal with background subtraction of cells only negative control. ID_50_s were calculated using Graphpad Prism v8.1 using nonlinear curve fitting with a Hill Slope constraint of < 0.

### Live virus neutralization

Isolates of SARS-CoV2 USA-WA1/2020, B.1.617.2, or BA.1 were obtained through BEI Resources, NIAID, NIH and handled in the BSL3 facility at the Wistar Institute. To grow virus stocks, Vero cells were inoculated with 0.01 MOI virus in DMEM. Supernatant was then collected 3 days post infection. To titer virus, Vero cells were seeded in DMEM with 1% FBS at 20,000 cells/well overnight. Virus stocks were serially diluted and transferred to wells. Five days post infection, wells were scored + /- for presence of cytopathic effect (CPE) by visual examination; virus titer was calculated using Reed-Munch method [73]. For neutralization assay, Vero cells were seeded at 20,000 cells/well overnight. Sera were heat inactivated for 30 min at 56ºC. Sera were then serially diluted in DMEM with 1% FBS and 1% P/S and incubated for 1 hr at room temperature with 300 TCID_50_/mL virus. This solution was then transferred to the Vero cells and scored five days post-infection for CPE. Neutralization titers were calculated using the Reed-Munch method.

### SARS-CoV-2 challenge

K18-hACE2 mice were immunized and shipped to the Public Health Agency of Canada (PHAC) for infection with Delta and BA.2 variants. Mice were anaesthetized with inhaled isoflurane and give 10^5^ TCID_50_ diluted in plain media via intranasal instillation (50 μL total, 25 per nare). And that they were monitored daily for signs of disease. A 20% weight loss cutoff and/or ataxia, paralysis, other neurological signs were used as clinical endpoints for euthanasia. A subset of mice in each group were euthanized at specified time points post-infection for analyses of lung pathology and viral load.

For pathology, tissues were fixed in 10% neutral phosphate buffered formalin, routinely processed, sectioned at 5 μm and stained with hematoxylin and eosin (HE) for histopathologic examination. Paraffin tissue sections were quenched for 10 min in aqueous 3% hydrogen peroxide. Epitope retrieval was performed using an in-house glycan retrieval solution in a Biocare Medical Decloaking Chamber (Biocare Medical, Pacheco, CA, USA). The primary antibody applied to the sections was SARS-CoV-2 (2019-nCoV) Nucleocapsid, Rabbit MAb (#40143-R019, Sino Biological Inc., Beijing, China) used at a 1:6000 dilution for thirty minutes. They were then visualized using a horse radish peroxidase labelled polymer, Envision + system (anti-rabbit) (Agilent, Santa Clara, CA, USA) and reacted with the chromogen diaminobenzidine (DAB). The sections were then counter stained with Gill’s hematoxylin.

Semi-quantitative lesion scoring was performed at day 4 post-challenge as follows: The percentage affected of each section examined was scored as 0 = no pathological changes, 1 = ≤25% of lung section affected, 2 = > 25% and ≤50% of lung section affected, 3 = > 50% and ≤75% of lung section affected and 4 = > 75% of lung section affected. Each section was assigned a severity score or 0 = no lesions, 1 = mild lesions, 2 = moderate lesions and 3 = severe lesions. A score of 0 = not present, 1 = present, and 2 = severe was given for the presence of type II pneumocyte hyperplasia. In total, a score was assigned for each section, which included percentage affected (/4), severity (/3) and histological features (/3) for a total score (/10).

### Plasmid design

RBD nanoparticle constructs were encoded in the pVAX vector as follows: IgE secretion tag followed by the LS-3 CD4^+^ T cell helper epitope, glycine-serine linker, SARS-CoV-2 spike positions 331–527 modified with glycans and select omicron positions, glycine serine linker, and *Helicobacter pylori* ferritin (PDB ID: 3BVE) positions 2–167 construct modified at N19Q to remove a PNGS [74]. Codons were optimized for human and mouse expression by Genscript. Research and clinical codon optimizations were confirmed for immunological equivalence.

RBD monomer constructs were encoded in the pVAX vector with an IgE secretion tag, SARS-CoV-2 spike positions 331–527, and a C-terminal 6x His tag. Monoclonal antibodies were synthesized as human IgG1 antibodies with heavy and light chains were encoded in separate pVAX plasmids. Plasmids were codon optimized for human and mouse by Genscript.

### Epitope surface and diversity analysis

To determine the most common residues that antibodies against class 1 and 2 antibodies make contact with, Pymol was used to identify RBD residues that are within 5 Å of the heavy or light chains for 39 total antibodies. for the following class 1 structures were used: B38 (PDB ID: 7BZ5), CC12.1 (PDB ID: 6XC2), CC12.3 (PDB ID: 6XC4), C105 (PDB ID: 6XCN), REGN10933 (PDB ID: 6XDG), CV30 (PDB ID: 6XE1), CV07–250 (PDB ID: 6XKQ), CB6 (PDB ID: 7C01), P2C-1F11 (PDB ID: 7CDI), BD-604 (PDB ID: 7CH4), BD-236 (PDB ID: 7CHB), COVA2–04 (PDB ID: 7JMO), C102(PDB ID: 7K8M), C1A-B12 (PDB ID: 7KFV), S728-1157 (PDB ID: 8D0Z), TH132 (PDB ID: 7YVH), TH281 (PDB ID: 7YVM), R40-1G8 (PDB ID: 7SC1), S2K146 (PDB ID: 7TAS), A23-58.1(PDB ID: 7LRS), P5C3 (PDB ID: 7P40). The following class 2 structures were used: C002 (PDB ID: 7K8S), 2–4 (PDB ID: 6XEY), CV07–270 (PDB ID: 6XKP), P2C-1A3 (PDB ID: 7CDJ), BD-368-2 (PDB ID: 7CHH), COVA2–39 (PDB ID: 7JMP), C104 (PDB ID: 7K8U), C119 (PDB ID: 7K8W), C121 (PDB ID: 7K8Y), S2M11 (PDB ID: 7K43), C144 (PDB ID: 7K90), NE12 (PDB ID: 7U9O), LY-CoV555 (PDB ID: 7KMG), BD23 (PDB ID: 7BYR), 5A6 (PDB ID: 7KQB), S2H13 (PDB ID: 7JV4), C110 (PDB ID: 7K8V), BG7–20 (PDB ID: 7M6H). The positions mutated in the variant RBDs that are within 5 Å of class 1 and 2 Fabs were used to generate Fig 2d. For the coloring of the RBD by B factor to indicate the most common residues contacted by antibodies, the insert-bfactor Python script from GitHub written by Axel Fischer https://github.com/axelfischer/insert-bfactor was used to make figures in Pymol.

For the SARS-CoV-2 variant analysis, the SARS-CoV-2 Variant Proportions data was downloaded from the Center for Disease Control and Prevention https://data.cdc.gov/Laboratory-Surveillance/SARS-CoV-2-Variant-Proportions/jr58-6ysp/about_data. The proportion of each of the variants over time was determined for data at the national level. Maximums of the proportions was determined. Variants that had a burden of at least 4 was defined in this paper as a variant of “high burden.” For each of the high burden variants, the Outbreak.Info database https://outbreak.info/compare-lineages was used for the prevalence of mutations in the variants. Defining mutations for each variant was defined as mutations that occur in >50% of sequenced viruses. For the B-factor sphere scaling, the number of variants with a mutation was categorized by frequency where the B factor was set to

For the analysis of sarbecovirus RBD diversity, unique, complete RBD sequences extracted from non-human, mammal sequences for sarbecovirus spikes from the Bacterial and Viral Bioinformatics Resource Center. Sequence with the name SARS-CoV-1 and -2 in their names were excluded from the analysis. An amino acid mμtisequence alignment with reference to SARS-CoV-2 RBD used to identify the percentage of viruses with deletions and the positions of these deletions on the SARS-CoV-2 spike to make figures in Pymol. Models of the Rssp7924 Yunnan (NCBI: MH615953) and Yunnan RP JCC9 2020 (NCBI: UUX91064) were created using the Google Collab AlphaFold2. Models were aligned to the SARS-CoV-2 RBD in Pymol to generate figures.

### Nanoparticle models

Models of each the RBD nanoparticles were created using three components: ferritin nanoparticle scaffold (PDB ID: 3BVE), GS linker, and USA-WA1/2020 SARS-CoV-2 RBD (PDB ID: 6M0J). The RBD structure was modified with asparagine and man9 sugars at all the sequons that were modified in designs using Rosetta’s Glycan Tree Modeler. The GS linker was generated using Google Collab AlphaFold2 [75]. A single subunit of the relevant combinations of antigen GS linker, and then copied to the biological assembly of ferritin to create 24-mers. The nanoparticle scaffold and RBDs are shown as surface filled models and GS linkers are shown in cartoon representation. Glycans are shown by spheres.

### *In vitro* production and purification

Nanoparticles, antigens, and antibodies were produced in the Expi293F transfection system (Thermo Fisher Scientific). Briefly, Expi293F cells were maintained in Expi293F media on a shaker at 37°C and 5% CO_2_. Expi293F cells transfected using ExpiFectamine (Gibco) according to manufacturer’s protocol. A 1:1 ratio of heavy chain to light chain plasmid were co-transfected according to the ExpiFectamine manufacturer’s protocol for monoclonal antibody production. Supernatant was harvested following immunogen or monoclonal antibody plasmid transfection 7 days post-transfection.

Nanoparticle supernatant was run via an Akta Pure system over an in-house column packed with Galnthus Nivalis Lectin Beads (Vector Labs). Following lectin purification, nanoparticle elution fractions were then pooled and concentrated, before size exclusion chromatography purification on an Superose 6 Increase column (Cytiva) on the AKTA Pure system under the flow of PBS + 0.02% sodium azide. Monomeric antigen supernatant was run over a HisTrap HP column (Cytiva) using an Akta Pure system. Following nickel purification, monomeric antigen elution fractions were then pooled and concentrated, before size exclusion chromatography purification on an Superose 200 Increase 10/300 column (Cytiva) on the AKTA Pure system under the flow of PBS + 0.02% sodium azide. Monoclonal antibodies transfection supernatant was purified by HiTrap Mab Select on the Akta Pure system. Fractions were pooled and concentrated then buffer exchanged into PBS.

### Serum IgG isolation and fab digestion

Serum samples were heat inactivated at 56°C for 1 hour. To isolate polyclonal IgG, sera was incubated by individual mouse on Nab Protein A/G columns (Thermo Scientific) end over end at 4°C for at least 40 hours. Columns were washed and IgG eluted according to kit protocol. An incubation of 10 minutes of end over end mixing of wash buffer and elution buffer was done between each centrifugation step for elution. Antibodies were concentrated and buffer exchanged into phosphate buffered saline (PBS) in a 30 kDa Amicon concentrator.

To digest antibodies, papain from papaya latex (Sigma Aldrich) was activated at 37°C for 15 minutes in 100mM Tris pH = 8, 2mM EDTA, 10 mM L-cysteine. Activated papain was added to IgG for 5 hours at 37°C. The digest was quenched with 0.03M iodoacetamide then concentrated and buffer exchanged into tris-buffered saline (TBS) in a 10kDa Amicon concentrator. Approximately 200–400 ug of this antibody digest mixture was incubated with 15 ug of hexapro spike trimer with disulfides introduced via V705C and T883C(76) [76]. Complexes were incubated for ~90 minutes at ambient temperature. Complexes were then purified on an Akta Pure system (GE Healthcare) on a Superose 6 Increase column under flow of TBS and detected with UV absorbance at 215nm. Fractions corresponding to complexes were concentrated to ~100 μL in a 10 kDa Amicon concentrator and immediately used for making nsEM grids.

### Negative stain electron microscopy

A total of 3 μL of purified proteins or EMPEM complex at ~0.025 mg/mL were adsorbed onto glow discharged carbon-coated Cu300 EM grids (Electron Microscopy Sciences). Grids were then washed 3 times with 6 μL of TBS buffer, if protein was not already in TBS. The grids were then stained with 3 μL of 2% w/v uranyl formate, blotted, and stained for 90 seconds with 3 μof the stain followed by a final blot. Micrographs were collected manually on a FEI Tecnai T12 microscope equipped with Oneview Gatan camera at 100kV and ~-1.6 µm defocus. EMPEM datasets were collected at 52,000x magnification and 2.356 Å/pixel.

Relion v5.0 was used for data processing. Laplacian of Guassian particle picking was performed to generate stacks of ~250,000–400,000 particles per complex. Particles were extracted and classified in 2D classification, 3D reconstruction, and 3D auto-refine. The reference for 3D classification and auto-refine was EMDB 25711. A subset of 3D classes with good Fab densities that were auto-refined were used for making composite figures on a spike model (EMDB 25711) using UCSF Chimera.

### Glycan occupancy and species analysis

Proteins (60 µg) were denatured with 9 M urea in 100 mM ammonium acetate, 20 mM glycine (pH 6) followed by reduction with 10 mM dithiothreitol (DTT) at 37°C for 1 h, and alkylation with 50 mM iodoacetamide (IAM) for 45 min at 37°C in the dark. Reduced and alkylated samples were buffer exchanged into 100 mM ammonium bicarbonate (pH 8), using 10 kDa Amicon concentrators, and samples were divided into six equal (10 µg) aliquots for proteolytic digestions. Three out of the six aliquots were digested with a modified triple digestion method (Arg-C/trypsin, elastase, and subtilisin) and combined into a single sample as previously described [77]. The fourth and fifth aliquots were digested with Arg-C/Trypsin and chymotrypsin [77]. The sixth aliquot was digested with α-Lytic Protease (New England Biolabs) at 1:20 (w/w) enzyme/substrate ratio in ammonium bicarbonate (pH 8) at 37°C for 16 h. After incubations, each sample was heat inactivated for 5 min at 100°C dried in a Speed-Vac centrifuge, And redissolved in ammonium acetate, pH 5.5.

The digested samples were de-glycosylated with Endo-H (New England Biolabs) at a concentration of 250 units per 10 µg with incubation at 37°C for 1 h, dried in a Speed-Vac, and re-dissolved in 100 mM ammonium bicarbonate (pH 8) prepared in O^18^-H_2_0 (Sigma). PNGase-F (lyophilized, Bulldog Bio), dissolved in O^18^-H_2_0, was added at 500 units per 10 µg and incubated at 37°C for 1 h followed by heat inactivation for 5 min at 100°C.

Digests (1 µg) were analyzed on an Orbitrap Astral (Thermo Fisher Scientific) in-line with a Vanquish Neo UHPLC system (Thermo Fisher Scientific). Peptides were injected onto an Acclaim PepMapTM 100 trap column (75 μm i.d. x 2 cm packed with 3 μm C18 resin; ThermoFisher Scientific) and separated by reversed-phase HPLC on a BEH C18 nanocapillary analytical column (75 μm i.d. x 25 cm, 1.7 μm particle size; Waters) using a 175 min gradient formed by 0.1% formic acid in water (mobile phase A) and acetonitrile (mobile phase B). Full MS spectra were acquired in the Orbitrap at 60,000 resolution with a scan range of 300–1800 m/z, automatic gain control (AGC) target of 3e6, and maximum injection time (max IT) of 50 ms. Data-dependent MS2 spectra were acquired in the Orbitrap for the most abundant ions (intensity threshold of 2e4) over 3 sec. at 15,000 resolution with an isolation width of 1.5 m/z, standard AGC, and max IT of 50ms. Unassigned and singly charged ions were rejected.

MS data were analyzed using Fragpipe, v 22.0 [78–80]. Tandem MS spectra were converted to mzML format using MSConvert from ProteoWizard, v 3.023045, and searched against the UniProt human sequence database (August 21, 2023) to which the RBD design sequences and common contaminants had been added. A target/decoy database containing reversed sequences appended to the target database was used to determine peptide probabilities and false discovery rate (FDR). For combined digest searches, no enzyme specificity was selected and a fixed carbamidomethylation modification on cysteine (+57.02146) was used. In addition, oxidation (+15.9994, M), deamidation (+2.9883, N), GlcNAc (+203.0794, N), and N-terminal pyroglutamate formation (-17.0265, N) were considered as variable modifications. A cutoff of 1% FDR was used for peptide and protein identifications and a minimum glycosylation site localization probability of >0.75 was used [81,82].

Peptide abundances of RBD designs were summed for each of the four samples (1 triple protease digest + 3 individual digests) at each glycosite (i.e., peptide containing the consensus motif: N-X-S/T). Specifically, the intensities of peptides containing high mannose (N + 203.0794) and complex-type (N + 2.9883) modifications were summed for each glycosite, and the unoccupied proportion was determined by summing the intensities of unmodified peptides that had an identical glycosylated peptide sequence. Total abundances were determined by summing each glycosylation state (high-mannose, complex, and unoccupied) across the four digest samples for each site. The proportion of each glycosylation state was calculated as the total abundance of each glycosylation state divided by the total abundance for each glycosite.

### Statistical analyses

Statistical analyses were performed in Graphpad Prism v10.0. For comparison between individual groups, unpaired two-tailed Student t-tests were performed. For comparisons between groups over time, two-way ANOVAs were performed. For comparisons of survival curves, Mantel-Cox tests were performed.

## Supporting information

S1 Fig Additional immunogenicity characterization of USA-WA1/2020 based nanoparticle vaccine construct. A) Binding titers to series of ancestral variants following single, 2 μg immunization of DNA encoding RBD g5.1 24mer. B) Scoring of pathology of IHC stained lung in a subset of B.1.617.2 challenged mice at day 4 post-challenge (n = 4 mice/group). C) Representative lung images of K18-hACE mice that were B.1.617.2 challenged mice at day 4 post-challenge. D) Binding endpoint titers of RBD g5.1 24mer or RBD monomer vs USA-WA1/2020 and BA.1 RBD antigens after 2ug immunization (n = 5 mice/group). E) Pseudovirus neutralization of RBD g5.1 24mer vs USA/WA1/2020 and BA.1 (n = 5 mice/group). For A, data was generated using pooled samples, so no statistical tests were run. For B, differences between pathology was assessed by Kruskal-Wallis tests followed by a post hoc Dunn’s analysis. For D and E, differences were assessed by Šídák multiple comparisons. * p < 0.05, ** p < 0.01, *** p < 0.001.
(PDF)

S2 Fig Analysis of sarbecovirus RBDs. Non-human mammalian non-classified sarbecovirus and SARS-related sarbecovirus RBDs were extracted from spike sequences from the Bacterial and Viral Bioinformatics Resource Center database. Complete and non-redundant RBD sequences were aligned to the SARS-CoV-2 RBD for analysis of insertions and deletions. A) The deletions are labeled relative to the USA-WA1/2020 numbering (PDB ID: 6M0J). A total of 102 sequences were used for this analysis and excluded any SARS-CoV-2 sequences. AlphaFold models of sarbecovirus RBDs were generated and aligned to the SARS-CoV-2 RBD structure. Examples of models of sarbecovirus RBDs containing deletions in the B) 444–448 and C) 472–490 loops are indicated by colored cartoons compared to SARS-CoV-2 RBD in gray.
(PDF)

S3 Fig Additional in vitro characterization. Mass spectrometry analysis of glycan occupancy and species at each of the designed glycan positions.
(PDF)

S4 Fig Additional immune characterization. Pooled A) pseudovirus and B) live virus neutralization of RBD 4mut g5.1 24mer vs. RBD g5.1 24mer following 10 μg immunization. Data were generated using pooled samples (n = 5 mice/group), so no statistical tests were run.
(PDF)

S5 Fig Omicron breadth in spike antigen experienced BALB/c mice. For A-B, immunization scheme described in Fig 5a; in brief, mice are experienced with DNA encoded D614G spike (n = 5 mice/group). Sera 3 weeks post boost was assessed for A) binding to a panel of Omicron RBDs, and B) pseudovirus neutralization of XBB. Labels denote boosting group after DNA spike prime. LOD is limit of detection. For C-D, immunization scheme described in Fig 5e; in brief, mice are mRNA-LNP spike experience (n = 5 mice/group). Sera 3 weeks post boost was assessed for C) binding to a panel of Omicron RBDs, and D) pseudovirus neutralization of XBB. Labels denote boosting group after mRNA-LNP spike prime. LOD is limit of detection. For A-D, differences in vaccine responses were assessed by Kruskal-Wallis tests followed by a post hoc Dunn’s analysis.
(PDF)

S1 Table Prevalence of mutation and variants. CDC variant proportion database entire U.S. dataset was sorted by variant over time. Variants with a reported proportion of 4 for at least one reported week were analyzed for mutations in each variant. In the case of identical RBD sequences, only the variant with the higher maximum variant burden is included. The percent of sequenced variants containing mutations are shown as a percent according to Outbreak Info database. Mutations in the majority of viruses of a given variant are colored red and indicated as mutation relative to USA-WA1/2020 virus. The relative maximum burden of each variant according to US-wide data from the CDC is shown as the maximum proportion and colored on gray scale where darker shades indicate greater burden of the variant. Residues are categorized by epitope based on which residues are within 5A of a representative Fabs for class1-B38 (7BZ5), class2- C002 (7K8S), class 3-S309 (6WPT), class 4-CR3022 (6YOR). Mutations highlighted in yellow are incorporated in 4mut design. ND = not detected in sequenced viruses of the variant.
(DOCX)
