# Correction: Immune-focused RBD nanoparticles induce cross-reactive, RBS-directed responses capable of variant-resistant SARS-CoV-2 neutralization

**DOI:** 10.1371/journal.ppat.1014593

**Published:** 2026-09-14

**Authors:** Kylie M. Konrath, Kevin Liaw, Nicholas J. Tursi, Madison E. McCanna, Bryce M. Warner, Ebony N. Gary, Xizhou Zhu, Cory Livingston, Cara Monastra, Drew Frase, Neethu Chokkalingam, Alana Huynh, Nicholas Shupin, Kelly Bayruns, Sinja Kriete, Amber Kim, Joyce Park, Robert Vendramelli, Thang Truong, Kevin Tierney, Kimberly Azaransky, Estella Moffat, Carissa Embury-Hyatt, Lynn A. Beer, Oreoluwa Solanke, Susanne N. Walker, Richa Kalia, Hsin-Yao Tang, Laurent M. P. F. Humeau, Trevor R. F. Smith, Darwyn Kobasa, David B. Weiner, Daniel W. Kulp

There are number of errors in the legends for Fig 1 and Fig 3. Please see the complete, correct Fig 1 and Fig 3 legend here.

S1 Fig legend is incorrect. It can be viewed below.

## Supporting information

S1 Fig Additional immunogenicity characterization of USA-WA1/2020 based nanoparticle vaccine construct. A) Binding titers to series of ancestral variants following single, 2 μg immunization of DNA encoding RBD g5.1 24mer. B) Scoring of pathology of IHC stained lung in a subset of B.1.617.2 challenged mice at day 4 post-challenge (n = 4 mice/group). C) Representative lung images of K18-hACE mice that were B.1.617.2 challenged mice at day 4 post-challenge. D) Average weight loss curves from B.1.617.2 challenge by group. For A, data was generated using pooled samples, so no statistical tests were run. For B, differences between pathology were assessed by Kruskal-Wallis tests followed by a post hoc Dunn’s analysis. For D, differences between the average weight by group were assessed by a Dunnett’s multiple comparisons test for the first four days (before succumbing to infection and the mice per group change * p < 0.05, ** p < 0.01, *** p < 0.001.
(TIFF)

**Fig 1:**
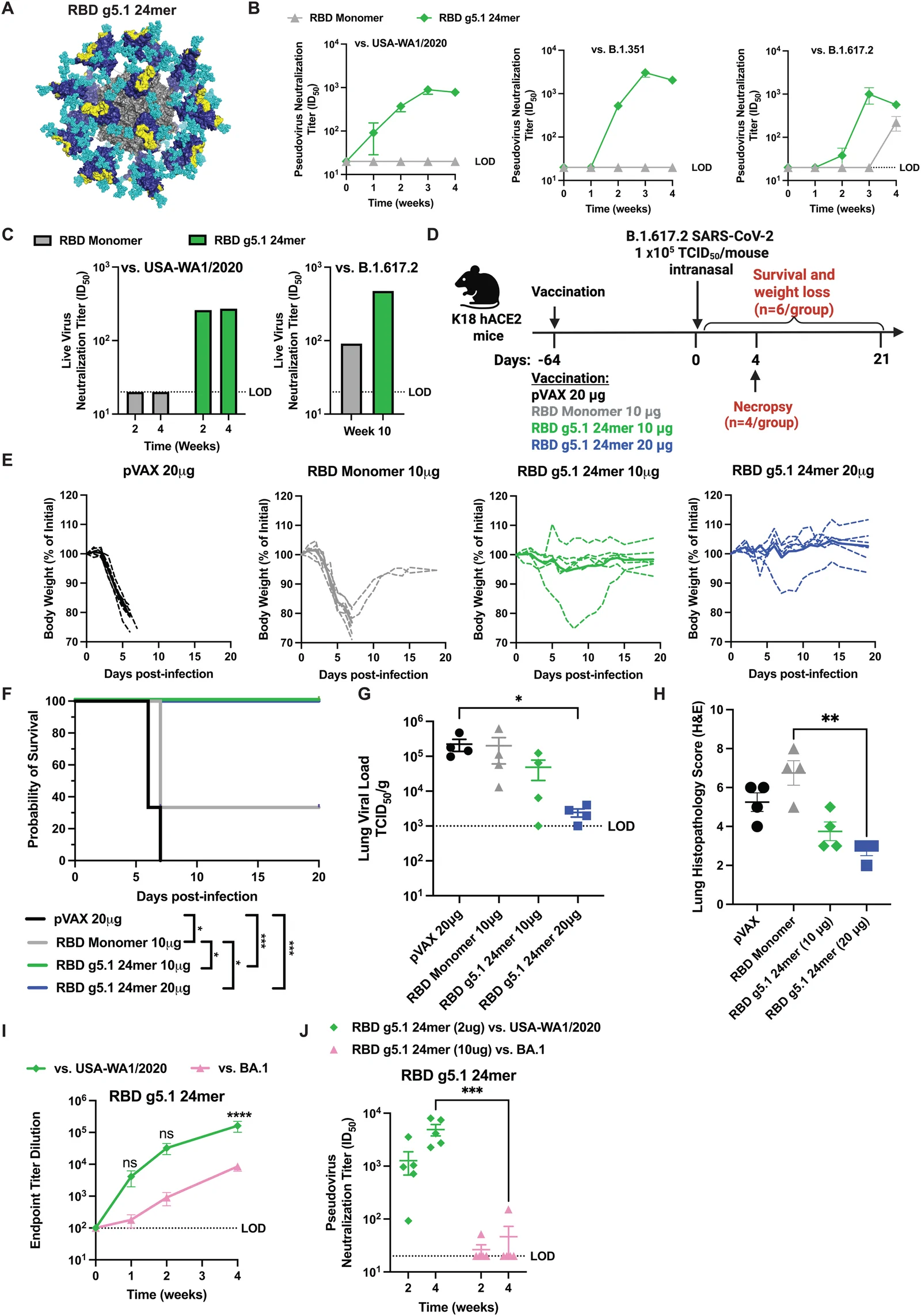
USA-WA1/2020 based RBD g5.1 24mer design induces cross-reactive responses through early SARS-CoV-2 variants and protects B.1.617.2 variant. A) Model of RBD g5.1 24mer nanoparticle where the surfaces for RBD, RBS, and ferritin scaffold are shown by surfaced colored dark blue, yellow, and gray respectively. Glycans are in cyan spheres. B) Pseudovirus endpoint titers to variant viruses of sera and C) live virus neutralization of sera from mice after single 2ug immunization (n = 5 mice/group pooled sera). Data in B and C were generated using pooled samples, so no statistical tests were run. D) Immunization scheme for in vivo lethal B.1.617.2 (Delta) variant challenge, with single immunization at 10ug or 20ug, followed 64 days after by 1x105 TCID50 intranasal B.1.617.2 challenge for data in E-H (n = 10 mice/group). Graphic was made using BioRender. E) Weight loss curves from B.1.617.2 challenge. Individual mice represented by dashed lie and solid line is the average. F) Survival was monitored post-challenge. Survival was compared by Mantel-Cox log-rank test. G) Viral loads in lung tissue four days post-challenge and differences analyzed by Kruskal-Wallis test followed by post hoc Dunn’s analysis. H) Pathology scoring of H&E stained lung in challenged mice in subset of mice at day 4 post-challenge and compared by Kruskal-Wallis test followed by a post hoc Dunn’s analysis. I) Binding endpoint titers of RBD g5.1 24mer or RBD monomer vs USA-WA1/2020 and BA.1 RBD antigens after 2ug immunization (n = 5 mice/group). J) Pseudovirus neutralization of RBD g5.1 24mer vs USA/WA1/2020 and BA.1 (n = 5 mice/group). For I and J, differences were assessed by Šídák multiple comparisons. * p < 0.05, ** p < 0.01, *** p < 0.001.

**Fig 3:**
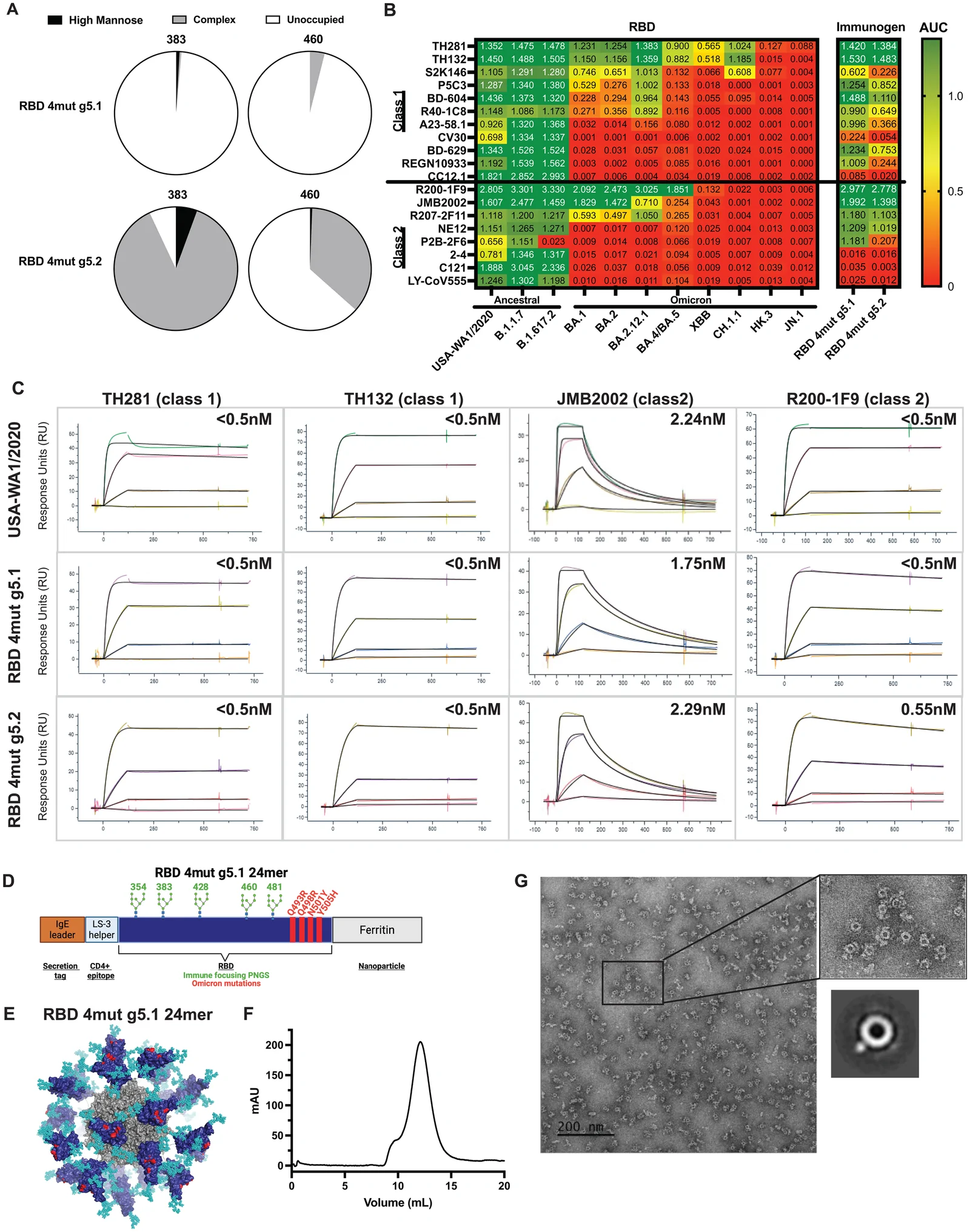
In vitro characterization of redesigned immunogens redesigned for cross-reactivity to ancestral and Omicron strains. A) Mass spectrometry analysis of glycan occupancy and species at each of the designed glycan positions. B) Heatmap of AUCs determined by ELISA for class 1 and 2 antibodies to immunogens and SARS-CoV-2 RBD antigens. C) Sensograms of most cross-reactive antibodies. KDs are corrected for glycan mass and occupancy and are indicated in the top right of each sensogram. D) Plasmid design of RBD 4mut g5.1 24mer nanoparticle where the secretion tag, CD4 + helper epitope, RBD, 4 mutations of BA.1, and ferritin scaffold are shown by orange, light blue, dark blue, red, and gray respectively. Glycans are indicated with cartoon. E) Model of RBD 4mut g5.1 24mer nanoparticle where the surfaces for RBD, 4 mutations of BA.1, and ferritin scaffold are shown by surfaced colored dark blue, red, and gray respectively. Glycans are in cyan spheres. F) Size exclusion chromatography trace of RBD 4mut g5.2 24mer. G) On the left, a negative stain electron micrograph of RBD 4mut g5.2 24mer with a zoomed in view of nanoparticles. A representative 2D class average of the nanoparticle is to the bottom right.
