# Supplementary figures and images for: Immune-focused RBD nanoparticles induce cross-reactive, RBS-directed responses capable of variant-resistant SARS-CoV-2 neutralization
Source: PLoS Pathog. 2026 Feb 19;22(2):e1013905. doi: 10.1371/journal.ppat.1013905 (PMC13120701; doi:10.1371/journal.ppat.1013905)

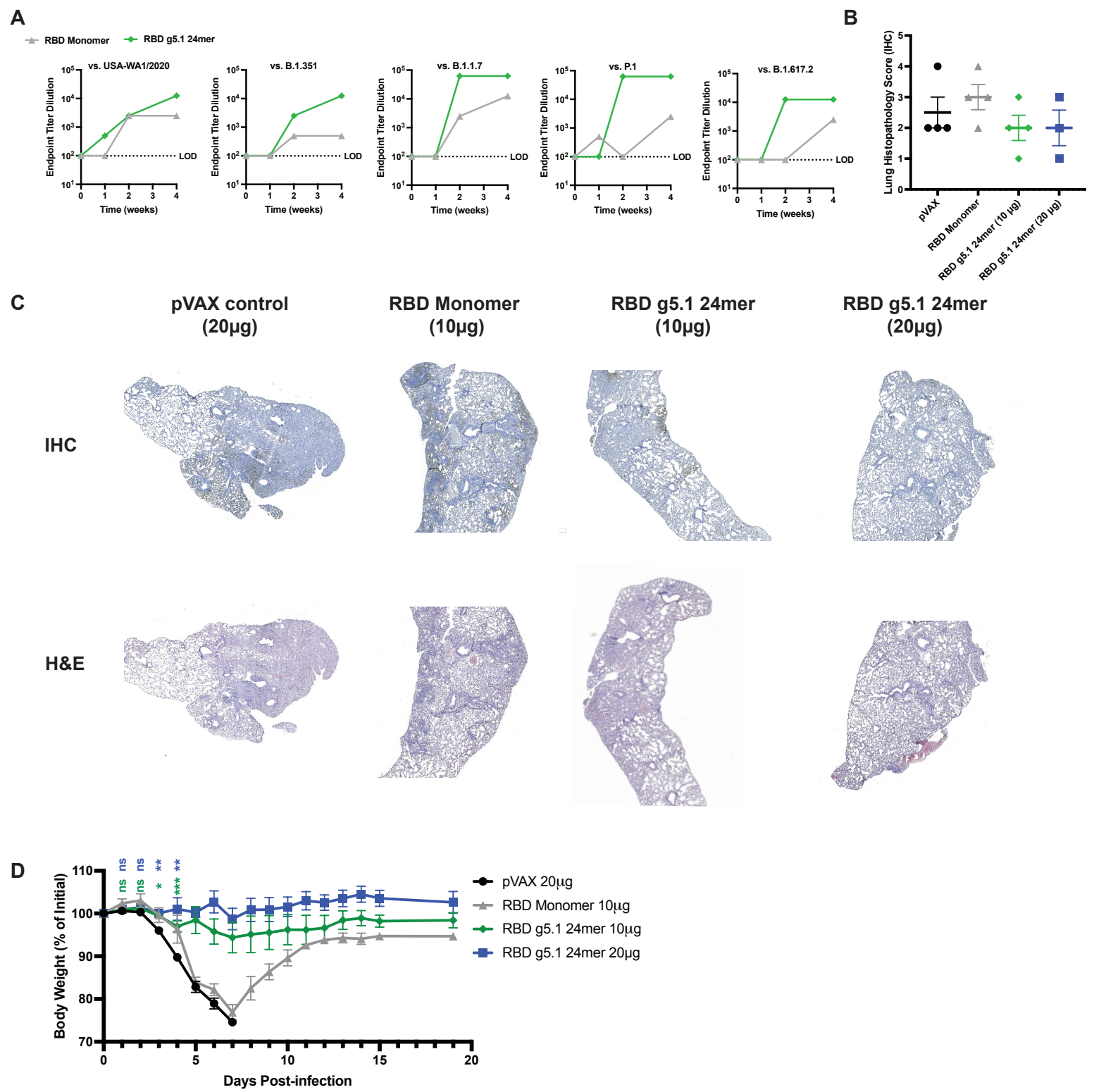

Supplement: S1 Fig — A) Binding titers to series of ancestral variants following single, 2 μg immunization of DNA encoding RBD g5.1 24mer. B) Scoring of pathology of IHC stained lung in a subset of B.1.617.2 challenged mice at day 4 post-challenge (n = 4 mice/group). C) Representative lung images of K18-hACE mice that were B.1.617.2 challenged mice at day 4 post-challenge. D) Binding endpoint titers of RBD g5.1 24mer or RBD monomer vs USA-WA1/2020 and BA.1 RBD antigens after 2ug immunization (n = 5 mice/group). E) Pseudovirus neutralization of RBD g5.1 24mer vs USA/WA1/2020 and BA.1 (n = 5 mice/group). For A, data was generated using pooled samples, so no statistical tests were run. For B, differences between pathology was assessed by Kruskal-Wallis tests followed by a post hoc Dunn’s analysis. For D and E, differences were assessed by Šídák multiple comparisons. * p < 0.05, ** p < 0.01, *** p < 0.001. (PDF) [file ppat.1013905.s001.pdf]

**A**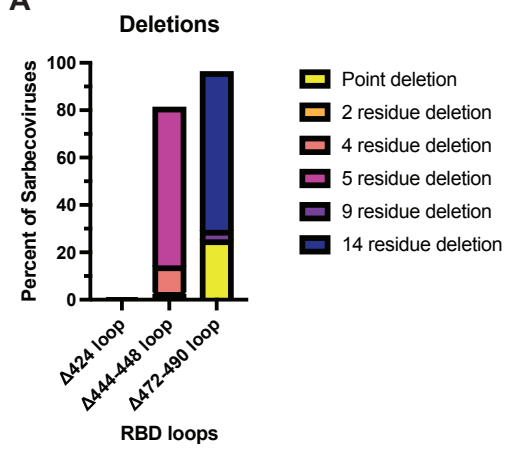**B**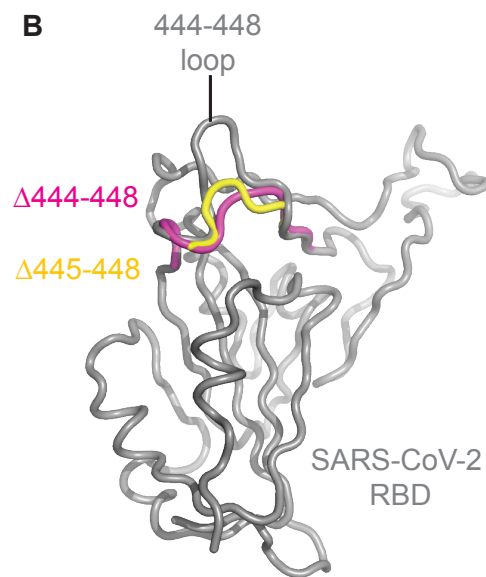**C**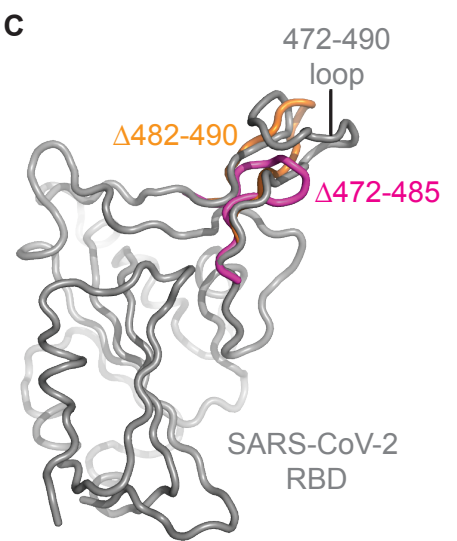

Supplement: S2 Fig — Non-human mammalian non-classified sarbecovirus and SARS-related sarbecovirus RBDs were extracted from spike sequences from the Bacterial and Viral Bioinformatics Resource Center database. Complete and non-redundant RBD sequences were aligned to the SARS-CoV-2 RBD for analysis of insertions and deletions. A) The deletions are labeled relative to the USA-WA1/2020 numbering (PDB ID: 6M0J). A total of 102 sequences were used for this analysis and excluded any SARS-CoV-2 sequences. AlphaFold models of sarbecovirus RBDs were generated and aligned to the SARS-CoV-2 RBD structure. Examples of models of sarbecovirus RBDs containing deletions in the B) 444–448 and C) 472–490 loops are indicated by colored cartoons compared to SARS-CoV-2 RBD in gray. (PDF) [file ppat.1013905.s002.pdf]

**A****RBD 4mut g5.1**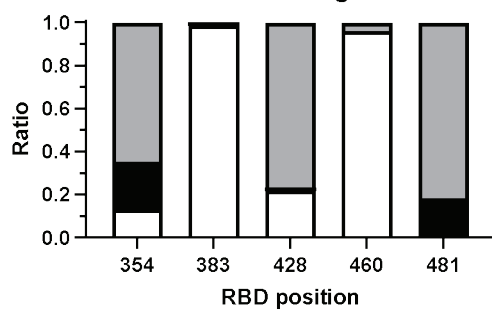**RBD 4mut g5.2**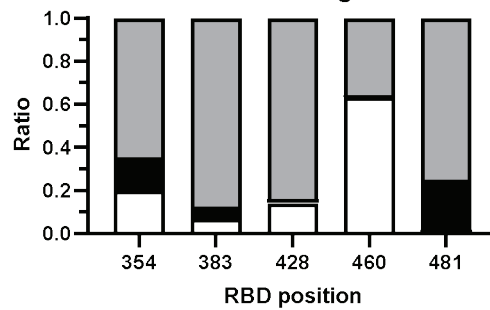**B**

■ RBD 4mut g5.1 24mer

▲ RBD 4mut g5.2 24mer

**Week 4**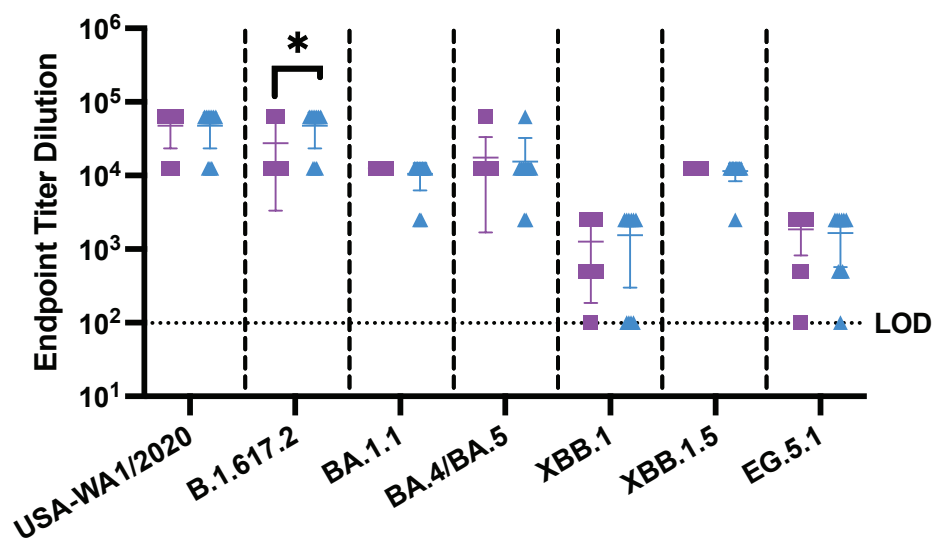**C**

■ RBD 4mut g5.1 24mer

▲ RBD 4mut g5.2 24mer

**Week 4**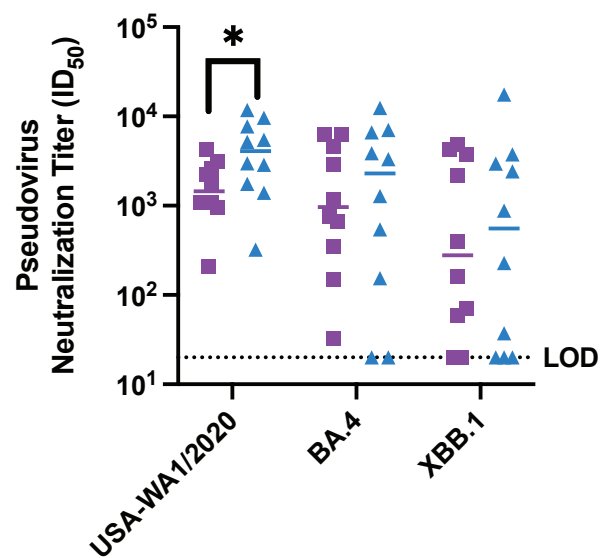

Supplement: S3 Fig — Mass spectrometry analysis of glycan occupancy and species at each of the designed glycan positions. (PDF) [file ppat.1013905.s003.pdf]

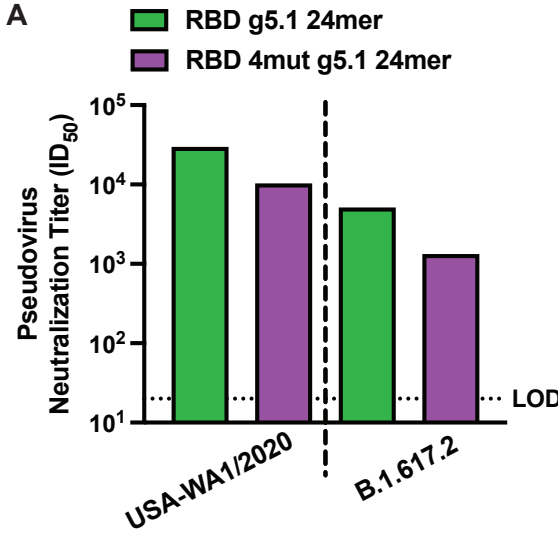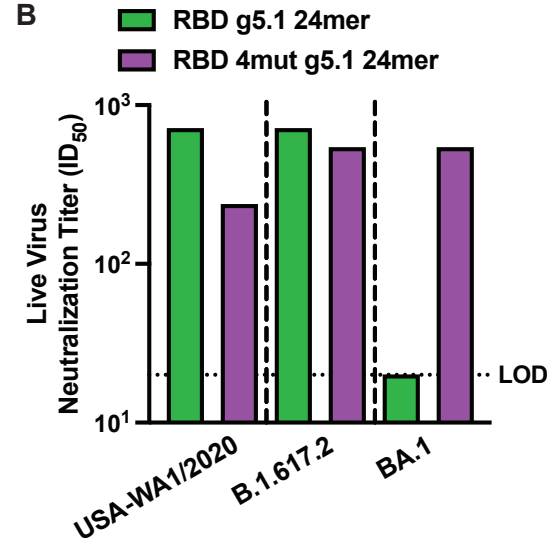

Supplement: S4 Fig — Pooled A) pseudovirus and B) live virus neutralization of RBD 4mut g5.1 24mer vs. RBD g5.1 24mer following 10 μg immunization. Data were generated using pooled samples (n = 5 mice/group), so no statistical tests were run. (PDF) [file ppat.1013905.s004.pdf]
