# Supplementary material for: Immune-focused RBD nanoparticles induce cross-reactive, RBS-directed responses capable of variant-resistant SARS-CoV-2 neutralization
Source: PLoS Pathog. 2026 Feb 19;22(2):e1013905. doi: 10.1371/journal.ppat.1013905 (PMC13120701; doi:10.1371/journal.ppat.1013905)
Supplement: S5 Fig — For A-B, immunization scheme described in Fig 5a; in brief, mice are experienced with DNA encoded D614G spike (n = 5 mice/group). Sera 3 weeks post boost was assessed for A) binding to a panel of Omicron RBDs, and B) pseudovirus neutralization of XBB. Labels denote boosting group after DNA spike prime. LOD is limit of detection. For C-D, immunization scheme described in Fig 5e; in brief, mice are mRNA-LNP spike experience (n = 5 mice/group). Sera 3 weeks post boost was assessed for C) binding to a panel of Omicron RBDs, and D) pseudovirus neutralization of XBB. Labels denote boosting group after mRNA-LNP spike prime. LOD is limit of detection. For A-D, differences in vaccine responses were assessed by Kruskal-Wallis tests followed by a post hoc Dunn’s analysis. (PDF) [file ppat.1013905.s005.pdf]

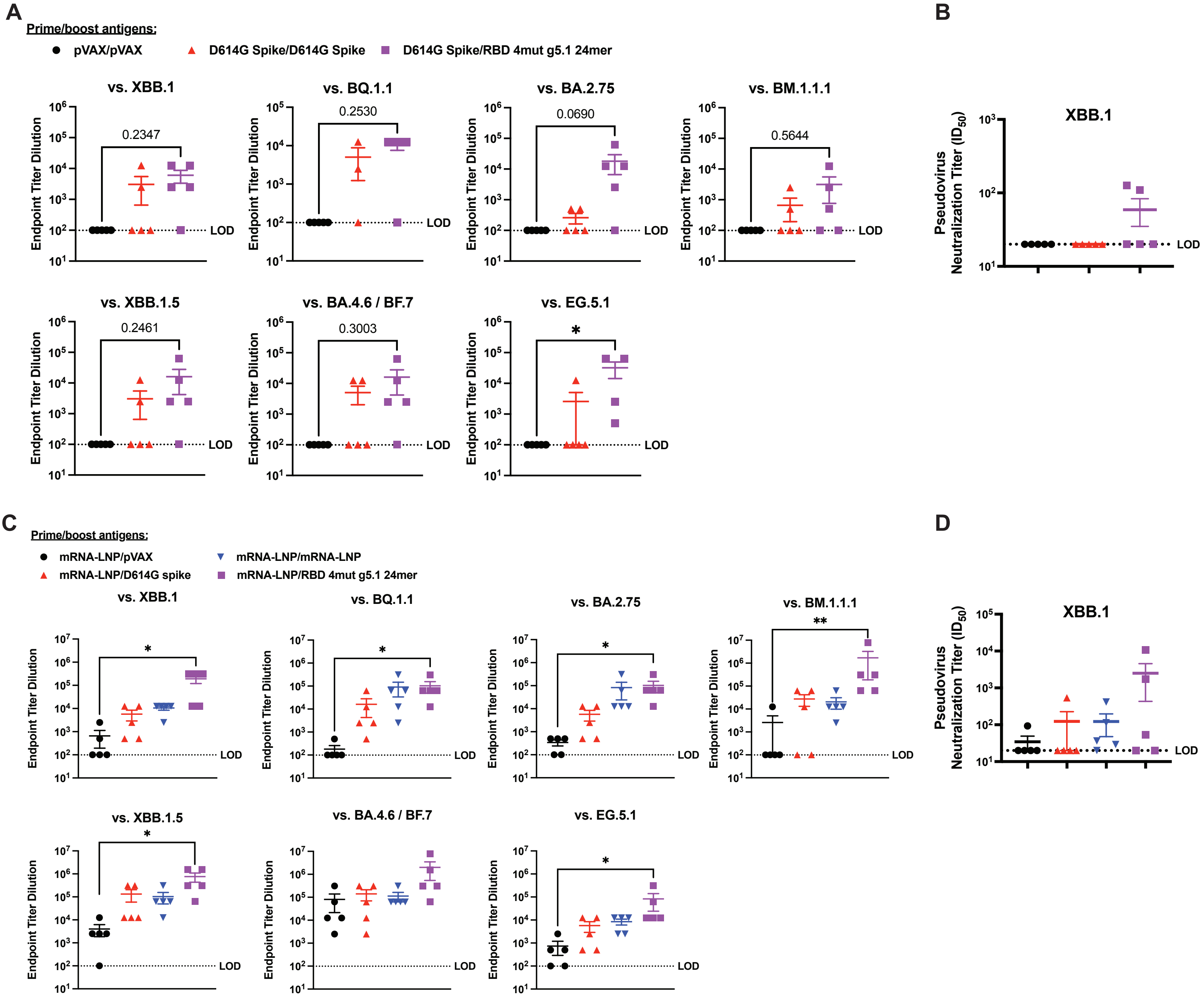
A
Prime/boost antigens:
pVAX/pVAX
D614G Spike/D614G Spike
D614G Spike/RBD 4mut g5.1 24mer
vs. XBB.1
0.2347
vs. BQ.1.1
0.2530
vs. BA.2.75
0.0690
vs. BM.1.1.1
0.5644
vs. XBB.1.5
0.2461
vs. BA.4.6 / BF.7
0.3003
vs. EG.5.1
*
Endpoint Titer Dilution
LOD
B
XBB.1
Pseudovirus Neutralization Titer (ID50)
LOD
C
Prime/boost antigens:
mRNA-LNP/pVAX
mRNA-LNP/D614G spike
mRNA-LNP/mRNA-LNP
mRNA-LNP/RBD 4mut g5.1 24mer
vs. XBB.1
*
vs. BQ.1.1
*
vs. BA.2.75
*
vs. BM.1.1.1
**
vs. XBB.1.5
*
vs. BA.4.6 / BF.7
vs. EG.5.1
*
Endpoint Titer Dilution
LOD
D
XBB.1
Pseudovirus Neutralization Titer (ID50)
LOD
